# Lower gestational age is associated with lower cortical volume and cognitive and educational performance in adolescence

**DOI:** 10.1186/s12916-022-02627-3

**Published:** 2022-11-03

**Authors:** Qing Ma, Hui Wang, Edmund T. Rolls, Shitong Xiang, Jiong Li, Yuzhu Li, Qiongjie Zhou, Wei Cheng, Fei Li

**Affiliations:** 1 Department of Neurology, Huashan Hospital, Institute of Science and Technology for Brain-Inspired Intelligence, State Key Laboratory of Medical Neurobiology and MOE Frontiers Center for Brain Science, Fudan University, Shanghai, 200433 China; 2 Key Laboratory of Computational Neuroscience and Brain-Inspired Intelligence, Fudan University, Ministry of Education, Shanghai, 200433 China; 3 Department of Developmental and Behavioral Pediatric & Child Primary Care/MOE-Shanghai Key Laboratory of Children’s Environmental Health, Xin Hua Hospital Affiliated to Shanghai Jiao Tong University School of Medicine, Shanghai, 200082 China; 4 Department of Computer Science, University of Warwick, Coventry CV4 7AL Conventry, UK; 5 Oxford Centre for Computational Neuroscience, Oxford, UK; 6 Department of Clinical Medicine, Aarhus University, Aarhus, 8000 Denmark; 7 Obstetrics and Gynecology Hospital of Fudan University, Shanghai, 200011 China; 8 Shanghai Key Laboratory of Female Reproductive Endocrine-Related Diseases, Shanghai, 200011 China; 9 Fudan ISTBI—ZJNU Algorithm Centre for Brain-inspired Intelligence, Zhejiang Normal University, Jinhua, 321004 China; 10 Shanghai Medical College and Zhongshan Hospital Immunotherapy Technology Transfer Center, Shanghai, 200032 China

**Keywords:** Gestational age, Cognitive performance, Neuroimaging, Cortical structure, Longitudinal development

## Abstract

**Background:**

Gestational age (GA) is associated with later cognition and behavior. However, it is unclear how specific cognitive domains and brain structural development varies with the stepwise change of gestational duration.

**Methods:**

This large-scale longitudinal cohort study analyzed 11,878 early adolescents’ brain volume maps at 9–10 years (baseline) and 5685 at 11–12 years (a 2-year follow-up) from the Adolescent Brain Cognitive Development (ABCD) study. According to gestational age, adolescents were divided into five categorical groups: ≤ 33 weeks, 34–35 weeks, 36 weeks, 37–39 weeks, and ≥ 40 weeks. The NIH Toolbox was used to estimate neurocognitive performance, including crystallized and fluid intelligence, which was measured for 11,878 adolescents at baseline with crystallized intelligence and relevant subscales obtained at 2-year follow-up (with participant numbers ranging from 6185 to 6310 depending on the cognitive domain). An additional large population-based cohort of 618,070 middle adolescents at ninth-grade (15–16 years) from the Danish national register was utilized to validate the association between gestational age and academic achievements. A linear mixed model was used to examine the group differences between gestational age and neurocognitive performance, school achievements, and grey matter volume. A mediation analysis was performed to examine whether brain structural volumes mediated the association between GA and neurocognition, followed with a longitudinal analysis to track the changes.

**Results:**

Significant group differences were found in all neurocognitive scores, school achievements, and twenty-five cortical regional volumes (*P* < 0.05, Bonferroni corrected). Specifically, lower gestational ages were associated with graded lower cognition and school achievements and with smaller brain volumes of the fronto-parieto-temporal, fusiform, cingulate, insula, postcentral, hippocampal, thalamic, and pallidal regions. These lower brain volumes mediated the association between gestational age and cognitive function (*P* = 1 × 10^−8^, *β* = 0.017, 95% CI: 0.007–0.028). Longitudinal analysis showed that compared to full term adolescents, preterm adolescents still had smaller brain volumes and crystallized intelligence scores at 11–12 years.

**Conclusions:**

These results emphasize the relationships between gestational age at birth and adolescents’ lower brain volume, and lower cognitive and educational performance, measured many years later when 9–10 and 11–12 years old. The study indicates the importance of early screening and close follow-up for neurocognitive and behavioral development for children and adolescents born with gestational ages that are even a little lower than full term.

**Supplementary Information:**

The online version contains supplementary material available at 10.1186/s12916-022-02627-3.

## Background

The prenatal period is a foundationally and universally critical phase for human brain development [1]. Thus, understanding how prenatal influences play out in later life has important implications for developmental neuroscience and for public health.

Brain volume and its developmental changes are related to cognitive functions [2]. The human brain shows an initial accelerating increase in gray matter volume from mid-gestation onwards, peaking around 6 years, and then slowing down and then shrinking roughly from late childhood into adulthood [2, 3]. During the third trimester, the human cerebral cortex undergoes especially rapid volumetric increase where there is marked growth with a fourfold increase and the emergence of sulci and gyri that is essential for human intelligence [4, 5]. Non-optimal development during these periods due to preterm birth may be associated with altered brain structure and function that persist during postnatal life [6–8] and higher morbidity and mortality [9, 10]. Therefore, understanding to what extent and how different gestational durations are associated with neurodevelopment in later life are essential to clarify our understanding of the association between prenatal factors and health outcomes.

Gestational age (GA) as a global proxy measure of in-utero progress of fetal development is closely related to health outcomes in children and adolescents [11, 12]. A substantial body of population-based evidence has shown that younger gestational age is associated with poorer cognitive and academic performance in children and adolescents [13–15]. Some neuroimaging studies indicated that gray matter volume or related structural measures (such as brain gyrification) may partially mediate the association between gestational age and cognitive function in children or adults [16–18]. Additionally, there are some longitudinal studies tracking the development of children’s and adolescents’ brain structures born with different gestational ages, with some evidence for “no catch-up” growth of brain volume in very preterm births compared to full-term births [19–21].

However, although not in all cases [17, 22], our current understanding of the relationship between GA, cognition and brain volume in adolescents largely stems from pairwise comparisons [21, 23, 24], such as comparing healthy controls born at full-term with those born very early preterm (< 26 gestational weeks) or very preterm (26 to 32 gestational weeks) or late preterm (34 to 36 gestational weeks). A definition of preterm birth as a dichotomous measure based on an arbitrary cut-off, rather than birth time at a specific fetal maturity level or at continuously graded gestational stages, limits our understanding of postnatal maturation. Most of the evidence on underlying brain structures mediating the association of prematurity and cognition is separate from studies focusing on preterm-related brain structural growth [17, 18, 21], and large-scale studies are needed that track the longitudinal development of specific brain structures linked to gestational age and cognition. Additionally, most previous investigations are limited to single-dimensional neurocognitive evaluation (e.g., IQ [21] or executive function [17]) or a small sample size [16, 19]. The current study focused on multi-dimensional cognitive characteristics including reading and language, working memory and attention, and processing speed. Moreover, gestational age may be associated with other confounding factors, such as birth weight and early Cesarean delivery due to unavoidable clinical complications, which may also affect the development of the offspring. This shows that there is a need for large-scale studies taking into account potential confounding variables to measure many aspects of brain structure, and of cognitive development into adolescence, and to relate these to continuously graded gestational age.

Given this background, the current investigation focused on clarifying the association between gestational age, the volumes of different brain regions, and dimensional measures of cognitive functions, using a large sample of 11,878 early adolescents aged 9–10 years with 2-year follow-up data from the Adolescent Brain Cognitive Development (ABCD) study [25] and validated by Danish cohort study of 618,070 adolescents at 15–16 years old [26]. The specific aims were as follows: (1) to assess whether shorter gestational duration is associated with a higher risk of poor cognitive function and lower volumes of some brain regions; (2) to examine whether the volume of some brain regions mediates the association between gestational age and cognition; (3) to examine whether different gestational ages have divergent growth trajectories for brain volumes and whether the association between gestational age and brain volume is still present in adolescence. It was hypothesized that shorter gestational duration is associated with poorer cognitive and academic performance in adolescents and that the underlying mechanisms could be related to long-lasting differences in brain structure in at least the temporal region because this is the latest brain area to mature during the third trimester.

### Role of the funding source

The funding sources had no role in the study design; in the collection, analysis, and interpretation of the data; in the writing of the report; and in the decision to submit the paper for publication.

## Methods

### Participants

**Fig. 1:**
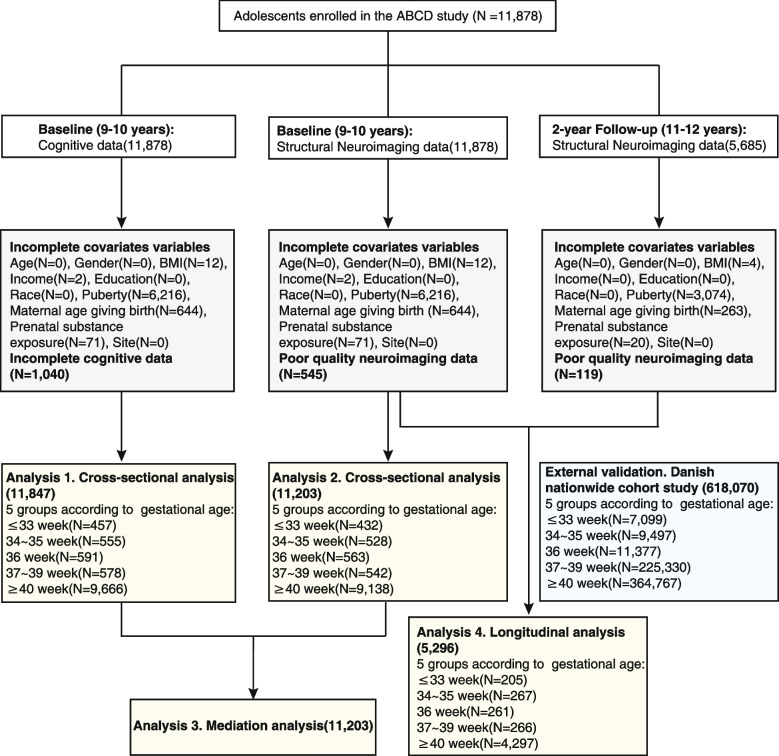
Flow diagram

The neuroimaging data and cognitive assessments used in this study were obtained from the Annual Curated Data Release 3.0 of the ABCD study (https://abcdstudy.org/scientists/data-sharing/), which is a large national-based longitudinal investigation of Adolescent Brain Cognitive Development across 21 research sites in the USA [27]. Participants and their parents or caregivers completed a set of visits consisting clinical interviews, surveys, neurocognitive tests, and neuroimaging. A visit for the population is ongoing every year for behavioral and cognitive assessments and every 2 years for neuroimaging scanning. In the current study, a total of 11,878 participants aged between 9 and 10 years were recruited at baseline. For follow-up data, we only included neuroimages, not cognitive performance which were largely missing. Participants with missing information of gestational age or blank cognitive assessments or whose magnetic resonance imaging scans did not pass quality control were excluded. A participant selection flowchart and demographics of the final enrolled participants in the neuroimaging analysis are provided in Fig. 1 and Table 1 respectively. The ABCD investigators obtained written and oral informed consent from parents and children, respectively [28]. More details of the subjects and the collection are provided at the ABCD website (https://abcdstudy.org/scientists/protocols/) and elsewhere [27, 29].

**Table 1:** Demographic characteristics of the ABCD participants enrolled for neuroimaging analysis

	**Baseline analysis (9-10 years)**						**Follow-up analysis (11-12 years)**					
	≤ 33 weeks (*N* = 432)	34 ~ 35 weeks (*N* = 528)	36 weeks (*N* = 563)	37 ~ 39 weeks (*N* = 542)	≥ 40 weeks (*N* = 9138)	*F*/*χ*^2^ (*P* value)	≤ 33 weeks (*N* = 266)	34 ~ 35 weeks (*N* = 205)	36 weeks (*N* = 267)	37 ~ 39 weeks (*N* = 261)	≥ 40 weeks (*N* = 4297)	*F*/*χ*^2^ (*P* value)
**Age (years) **^**a**^	10.0 (0.62)	10.0 (0.59)	10.0 (0.61)	10.0 (0.61)	9.9 (0.63)	13.1 (1.1 × 10^−10^)	12.0 (0.63)	12.0 (0.64)	12.1 (0.64)	12.0 (0.63)	11.9 (0.64)	8.5 (7.6 × 10^−7^)
**Gender **^**b**^						5.5 (0.24)						3.16 (0.53)
Female	201	237	284	245	4399		111	93	116	121	1999	
Male	231	291	279	297	4739		155	112	151	140	2298	
**BMI **^**a**^	18.7 (4.32)	17.9 (3.83)	18.8 (4.07)	18.9 (3.99)	18.8 (4.18)	5.7 (0.001)	18.6 (3.71)	18.1 (3.87)	18.0 (4.0)	19.1 (4.27)	18.7 (4.00)	3.48 (0.008)
**Total parent income **^**b**^						48.8 (6.8 × 10^−8^)						33.4 (5.3 × 10^−5^)
< $50,000 (low)	127	85	135	119	2530		53	53	36	58	1153	
$50,000–100,000 (middle)	108	148	162	137	2359		72	59	87	85	1171	
> $100,000 (high)	197	295	266	286	4249		141	93	144	118	1973	
**Parents Education **^**a**^	16.2 (2.66)	17.2 (2.17)	16.8 (2.46)	16.7 (2.66)	16.6 (2.81)	9.52 (1.2 × 10^−7^)	16.6 (2.74)	16.4 (2.40)	17.3 (2.00)	16.7 (2.45)	16.7 (2.70)	3.73 (0.005)
**Race **^**b**^						42.2 (3.1 × 10^−5^)						21.3 (0.05)
White	302	406	389	409	6183		32	26	30	31	609	
Black	68	63	99	51	1503		213	154	212	197	3077	
Indian	2	2	2	3	52		20	25	23	31	584	
Others	60	57	73	79	1400		1	0	2	2	27	
**Puberty **^**a**^	1.89 (0.68)	1.80 (0.57)	1.90 (0.59)	1.83 (0.56)	1.82 (0.62)	3.31 (0.01)	1.75 (0.53)	1.88 (0.63)	1.73 (0.54)	1.87 (0.56)	1.79 (0.61)	3.30 (0.01)
**Maternal age at birth **^**a**^	32.2 (7.22)	32.7 (6.58)	32.5 (7.53)	33.2 (8.17)	31.7 (8.39)	6.66 (2.4 × 10^−5^)	33.2 (7.05)	33.0 (6.84)	32.4 (6.29)	32.2 (7.06)	31.8 (8.08)	3.24 (0.01)
**Prenatal substance exposure before knowing of pregnancy **^**b**^						15.2 (0.51)						5.29 (0.99)
Tobacco	54	71	106	85	1179		46	18	30	50	536	
Marijuana	19	17	36	27	530		11	5	6	14	235	
Cocaine/crack	3	3	4	2	73		1	0	2	1	33	
Heroin/morphine	0	1	0	0	21		0	0	1	0	11	
Oxytocin	2	1	0	1	35		0	1	0	0	16	
**Prenatal substance exposure after knowing of pregnancy **^**b**^						13.7 (0.62)						14.5 (0.27)
Tobacco	21	28	40	32	445		17	9	13	14	184	
Marijuana	7	4	13	9	189		7	2	1	4	83	
Cocaine/crack	2	2	2	2	35		1	0	1	0	16	
Heroin/morphine	0	1	0	0	17		0	0	1	0	8	
Oxytocin	2	1	0	1	19		0	1	0	0	6	

*Abbreviations*: *BMI* body mass index (calculated as weight in kilograms divided by the square of height in meters)

^a^Data are presented as mean (standard deviation or percentage) of the measures

^b^Data are presented as number of participants

### Gestational age determination

Gestational age at birth was calculated based on the parent retrospective report on the following items: “Was the child born prematurely?” (devhx_12a_p) and “About how many weeks premature was the child when they were born?” (devhx_12_p). Considering that term adolescents defined in the second item include all adolescents born at full term defined in the first item and that the second item gives the specific number of premature weeks, we relied primarily on the second item to determine weeks of gestation. According to the answers, gestational age for term adolescents was defined as 40 weeks and preterm adolescents as 40 weeks minus the number of weeks premature. Participants were excluded if information was not provided (answering “Don't know’). The final gestational age ranges from less than 28 to 40 weeks. Referring to the grouping of gestational age in some previous research studies [12, 30, 31] and considering approximate matching of sample sizes, five exclusive groups were formed: adolescents born at less than 33 weeks, 34–35 weeks, 36 weeks, 37–39 weeks, and 40 weeks of gestation. We additionally compared the group differences in cognitive performance between each pair of gestational ages. The distribution of gestational age for neuroimaging analyses is provided in Fig. S1. Further, considering that grouping together < 28–33 weeks may not reflect the cognitive differences in this gestational age range, we divided this range of gestational age into ≤ 28 weeks and 29 to 33 weeks for validation.

### Cognitive assessment

Cognitive performance was measured using the fully-corrected composite *T* score within the NIH Toolbox which is a standardized battery of cognitive tests to estimate general intellectual functioning [32]. It incorporates performance from seven different tests to characterize two kinds of cognition: crystallized and fluid intelligence. The crystallized intelligence score summarizes scores from (1) the oral reading recognition test and (2) the picture vocabulary test. The fluid intelligence score summarizes scores from (3) the flanker inhibitory control and attention test, (4) the list-sorting working memory test, (5) the dimensional change card sort test, (6) the pattern comparison processing speed test, and (7) the picture sequence memory test. We also included three other kinds of cognitive measures for validation, including the Little Man Task for measuring visuospatial processing flexibility and attention, the Rey Auditory Verbal Learning Test for verbal learning and memory, and the Matrix Reasoning Task for measuring fluid intelligence and visuospatial reasoning.

### Neuroimaging

Magnetic resonance imaging (MRI) scanning was optimized and harmonized across the ABCD sites for 3-T scanners and preprocessing procedures were unified according to a pipeline performed by the ABCD team [33]. Cortical surface reconstruction and subcortical segmentation were processed through FreeSurfer v.5.3.0 on acquired T1-weighted anatomical scans by the ABCD team. The current study focused on brain cortical gray matter volume estimates using the Desikan-Killany Atlas [34] (34 regions per hemisphere) and subcortical volume estimates using the Automatic Subcortical Segmentation [35] (40 labels for ASEG regions). Image quality control was performed by the ABCD official team, and only the data recommended for use were included in our analysis. Detailed image preprocessing and quality control are shown in the Additional file 1 [27, 29, 33].

### Statistical analysis

#### Cross-sectional analysis

A linear mixed-effects model (LMM) was used to test the group difference of gestational age on the volumes of brain regions and cognitive performance, implemented using the MATLAB function *fitlme*. Dependent variables in the model included regional volumes and cognitive scores. The independent variable was the five gestational age groups. A sets of nuisance covariates to be regressed out were modeled as fixed effects, and the imaging site was accounted for random intercept parameters. The following were the covariates of no interest: adolescents’ age, sex, body mass index, family income, parental education year, race (coded as three-column dummy variables: white, black and American Indian), puberty scores, maternal age at delivery, and prenatal exposure to any kind of substance (tobacco, marijuana, cocaine/crack, heroin/morphine, oxycontin) before or after maternal knowledge of pregnancy. An *F*-statistic was computed using MATLAB’s *anova* function to determine the difference between the gestational age groups. Then, a specified comparison was carried out between each preterm GA group and the 40-week full-term group to test how brain volume and cognitive performance varied with differences in the number of gestational weeks. A *t*-statistic was obtained in this step by using *fitlme* with 40 weeks serving as the reference level. Test of significance for both *F*- and *t*-statistics were corrected for multiple comparison using Bonferroni correction at the 0.05 level. Partial eta-square *η*^2^_p_ and Cohen’s *d* values, representing the effect size of the group difference, were computed from the *F* statistic and *t* statistic respectively. The analysis was performed at both baseline (11,203 participants) and 2-year follow-up (5553 participants) for neuroimaging data and at baseline (11,847 participants) for all cognitive dimensions and at 2-year follow-up (number of participants ranging from 6185 to 6310) for some available cognitive domains.

#### Mediation analysis

This is to evaluate whether the covariance between gestational age and cognitive performance can be explained by the lower brain volumes identified for lower gestational ages. This procedure was performed using the Mediation Toolbox developed by Tor Wager’s group (https://github.com/canlab/MediationToolbox), which has been widely used in neuroimaging studies [36–38]. In the analysis, the independent (predictor) variable was gestational age ranging from 27 to 40 weeks, and the dependent (predicted) variable was cognitive score (the total, crystallized or fluid intelligence score). The proposed mediator (in the indirect path) was the mean cortical volume for regions identified as having group difference at baseline (Fig. 3A). We also took each individual regional volume as mediator and performed the same analysis (Table S4). Covariates used in the models were identical with those in the “ Cross-sectional analysis” section. The significance of the mediation was estimated by the bias-corrected bootstrap approach (with 10,000 random samplings), which has been proved to be more robust to nonnormality and has better type I error control than the causal steps method and the Sobel test [39]. All variables in the mediation analysis were based on baseline neuroimaging and cognitive data (11,203 participants) and were normalized to z-scores before calculations.

#### Longitudinal analysis

For regional volumes showing significant group differences at baseline, their developmental changes were measured 2 years later to detect whether adolescents have different developmental trajectories due to different gestational weeks. The longitudinal analysis was performed by a linear mixed model using the function *fitlme*. The dependent variables were the cortical volumes of significant brain regions and their average value. Independent variables were gestational age with five categorical groups and time with two-levels (baseline and 2-years follow-up). A set of the same nuisance covariates were the same as the “ Cross-sectional analysis” section and modeled as fixed effects, and the site was designed as random effect. Partial eta-square *η*^2^_p_, representing the effect size of the group-by-time interaction, was computed from the *F* statistic, with significance set as *P* < 0.05 by Bonferroni correction for multiple comparisons. The analysis was performed for 5296 participants who have both baseline and follow-up neuroimaging data.

### Confounding factors

Due to the fact that birth weight usually has a high correlation with gestational weeks [13], we excluded adolescents with extremely high or low birth weight relative to the expected gestational weeks and then reanalyzed the effect of gestational weeks on cortical volume. Both small and large for gestational age (SGA and LGA) were excluded, which was defined as birth weight more than two SDs below (above) the mean weight relative to that expected for sex and gestational age, according to the Canadian national dataset [40]. Then, because cesarean delivery is unspontaneous premature delivery often caused by clinical complications, which may have different underlying mechanisms from spontaneous prematurity [41], we removed these participants and then replicated the analysis in the population with spontaneous delivery only. Moreover, as socioeconomic factors are consistently considered to be closely related to the brain and cognitive development [42–44], we tested whether family income was related to the association between gestational age and cortical volume. The family income was classified as three levels, with low level of < $50,000, middle level of $50,000–100,000, and high level of > $100,000. Then, a group-by-income interaction analysis was conducted to detect any relation of the income difference to the association between gestational weeks and cortical volumes at baseline.

### External validation analysis using the Danish nationwide cohort study

Different from cognitive ability tests, academic achievement requires the integration of multiple cognitive domains and has practical educational significance because it reflects learning abilities in real society [45]. To clarify whether gestational age has a long-term association with mid-adolescents’ school achievements, we utilized a population-based cohort including 618,070 adolescents at the age of 15–16 years from the Danish national register (detailed characteristics see Table S1) [26, 36, 46–49]. In the Danish cohort, information on gestational age was obtained from the Danish Medical Birth Resister. The length of gestation was estimated by ultrasonography examination, last menstrual period, or clinical examination, which has been frequently used to estimate gestational age in previous studies [50, 51]. Examination grades in Danish and Mathematics, which consisted of five profile areas including oral, reading comprehension, spelling, problem solving and mathematical skills, were analyzed. The grades were standardized as *z* scores for each gestational age group (22 to 33, 34 to 35, 36, 37 to 39, and ≥ 40 gestational weeks). A multiple linear regression model was used to estimate the difference of school grades between each GA group and the reference group (GA ≥ 40 week). More details are provided in Additional file 1.

## Results

### Participant characteristics

This study evaluated 11,878 adolescents (5366 [47.9%] female) at baseline (9–10 years), with 5685 (2615 [46.0%] female) having 2-year follow-up data. Imaging data from 545 and 119 individuals were excluded respectively for the two time points because of failure to pass quality control measures. The final participants for baseline imaging analysis were divided into five exclusive groups according to gestational age, with 432, 528, 563, 542, and 9138 adolescents born at less than 33 weeks, 34–35 weeks, 36 weeks, 37–39 weeks, and 40 weeks (full term) of gestation, respectively. The demographics and participant selection procedures are presented in Table 1 and Fig. 1.

### Younger gestational age and lower cognitive function at 9–10 years and 2 years later

**Fig. 2:**
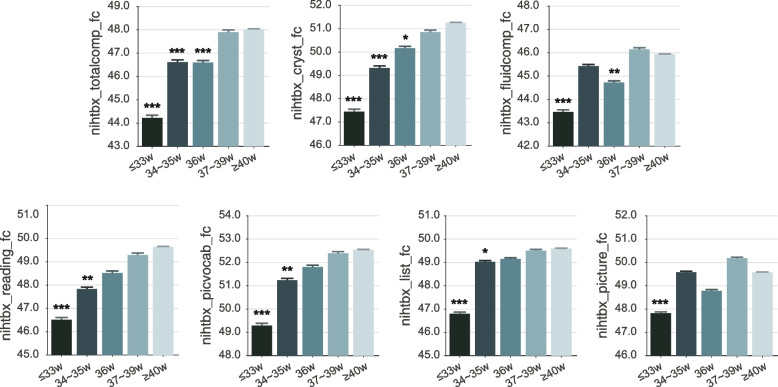
Significant group difference of gestational age on cognitive performance in 11,847 adolescents at baseline. All displayed cognitive measurements are corrected by Bonferroni multiple comparisons at 0.05 level in the linear mixed model analysis. The *Y*-axis is the cognitive score (from the abcd_tbss), and the error bar is the standard deviation. Asterisks indicate that the corresponding cognitive scores of each gestational age group differ from that in the 40-week gestational group at *P* < 0.001(***), *P* < 0.01(**), or *P* < 0.05(*) (all Bonferroni corrected). Age, sex, body mass index, family income, parental education year, race, puberty score, maternal age at delivery, and prenatal exposure to any kind of substance before or after maternal knowledge of pregnancy were regressed out in all analyses. Note: nihtbx_totalcomp_fc: Cognition Total Composite Score Fully-Corrected T-score; nihtbx_cryst_fc: Crystallized Composite Fully-Corrected T-score; nihtbx_fluidcomp_fc: Cognition Fluid Composite Fully-Corrected T-score; nihtbx_reading_fc: NIH Toolbox Oral Reading Recognition Test Age 3 + v2.0 Fully-Corrected T-score; nihtbx_picvocab_fc: NIH Toolbox Picture Vocabulary Test Age 3 + v2.0 Fully-Corrected T-score; nihtbx_list_fc: NIH Toolbox List Sorting Working Memory Test Age 7 + v2.0 Fully-Corrected T-score; nihtbx_picture_fc: NIH Toolbox Picture Sequence Memory Test Age 8 + Form A v2.0 Fully-Corrected T-score. The Fully-Corrected T-score in the NIH Toolbox Cognition Battery was a type of standardized score based on a score distribution that has a mean of 50 and a standard deviation of 10

Overall, differences of cognitive performances were associated with gestational age (GA) (*P* < 0.05 to *P* < 0.001, Bonferroni corrected; Fig. 2). A positive association was observed between GA and the total intelligence score (regression coefficient [*β*] = 0.40/week; 95% CI, 0.30–0.49/week), crystallized intelligence score (*β* = 0.40/week; 95% CI, 0.30–0.49/week) as well as fluid intelligence score (*β* = 0.26/week; 95% CI, 0.16–0.36/week). For every 1-week longer gestational duration, the total intelligence score, crystallized intelligence score, and fluid intelligence score increased by 0.8%, 0.8%, and 0.6%, respectively. Adolescents born at ≤ 33 week had an 8.6% decrease in total intelligence score compared with those at ≥ 40 week and a decline of 8.0% and 5.7% for crystallized intelligence score and fluid intelligence score (Fig. 2). Pair-wise comparison at each gestational age showed that the difference in cognitive scores increased as the time interval between gestational weeks got longer (Additional file 1: Fig. S2). Compared to adolescents born at ≥ 40 weeks, adolescents born at less than 33 weeks, 34–35 weeks, and 36 weeks showed significantly lower total intelligence cognitive scores (*P* < 0.05, Bonferroni corrected) (presented as effect size using Cohen’s *d* with 95% CI) (d =  − 0.44, − 0.56 to − 0.31; *d* =  − 0.16, − 0.25 to − 0.08; *d* =  − 0.14, − 0.22 to − 0.06) and crystallized intelligence score (*d* =  − 0.44, − 0.56 to − 0.31; *d* =  − 0.20, − 0.29 to − 0.12; *d* =  − 0.11, − 0.19 to − 0.02). For the fluid intelligence score, only adolescents of less than 33 weeks (*d* =  − 0.29, − 0.41 to − 0.16) and 36 weeks (*d* =  − 0.12, − 0.2 to − 0.04) showed significantly lower cognitive performance. Additionally, compared to adolescents born at ≥ 40 weeks, adolescents born at ≤ 28 weeks and 29 to 33 weeks also showed a lower total intelligence score (*d* =  − 0.39, − 0.65 to − 0.14; *d* =  − 0.30, − 0.4 to − 0.2), crystallized intelligence score (*d* =  − 0.42, − 0.67 to − 0.16; *d* =  − 0.31, − 0.41 to − 0.21) and fluid intelligence score (*d* =  − 0.39, − 0.65 to − 0.14; *d* =  − 0.19, − 0.36 to − 0.16). There were no significant differences in cognitive performance between adolescents born at ≤ 28 weeks and those born at 29 to 33 weeks (total intelligence score: *d* =  − 0.15, − 0.43 to 0.12; crystallized intelligence score: *d* =  − 0.06, − 0.34 to 0.21; fluid intelligence score: *d* =  − 0.14, − 0.41 to 0.13). Table S2 presents the statistical results for the subscales. Intriguingly, cognitive differences between gestational groups did not differ by sex (interaction *P* > 0.3). Further, lower cognitive performances on other measuring tools, including the Little Man Task, Rey Auditory Verbal Learning Test, and the Matrix Reasoning Task were associated with in a graded way with lower gestational age toward extremely preterm (as shown in Additional file 1: Table S3).

Similar results were observed at the age of 11–12, namely that differences of cognitive performance were associated with gestational age (GA) (*P* < 0.05 to *P* < 0.01, Bonferroni corrected; Table S4). A positive association was found between GA and the crystallized intelligence score (*β* = 0.37/week; 95% CI, 0.25–0.48/week). For every 1 week longer of gestational duration, the crystallized intelligence score was higher by 0.74%. Compared to adolescents born at 40 weeks, adolescents born at less than 33 weeks, 34–35 weeks, and 36 weeks showed significantly lower crystallized intelligence scores (*d* =  − 0.31, − 0.44 to − 0.18; *d* =  − 0.19, − 0.30 to − 0.08; *d* =  − 0.13, − 0.24 to − 0.02). For total and fluid intelligence scores, there were no results because of missing data. The statistical results on the sub-domains of cognitive abilities were presented in Additional file 1: Table S4.

### Younger gestational age is associated with lower volumes of brain regions at 9–10 years

**Fig. 3:**
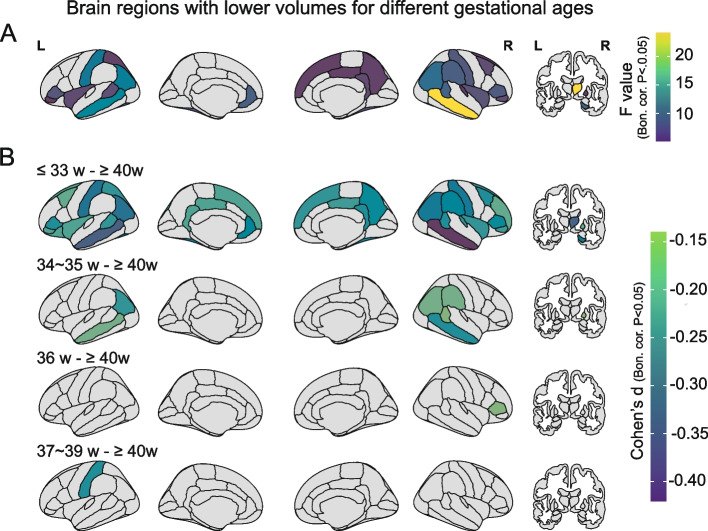
Significant group differences of brain volume for different gestational ages in 11,203 adolescents at baseline. Brain map parcellation is according to the Desikan-Killiany atlas and regions are outlined in black. **A** Significant group differences of brain volume for different gestational age measured by a one-way ANOVA following the linear mixed model. The color represents the *F* value (Bonferroni corrected, *P* < 0.05). **B** Pairwise comparisons of the volumes of brain regions for adolescents in each group of gestational age and those at 40 weeks with a statistical threshold. The color represents the Cohen’s *d* value (Bonferroni correction, *P* < 0.05). L: left, R: right; Bon. cor.: Bonferroni correction. The surface visualization was generated using the R package *ggseg* (https://github.com/ggseg/ggseg)

Lower cortical and subcortical volumes found within the five gestational groups were primarily located in fronto-parieto-temporal areas, the fusiform gyrus, cingulate cortex, insula, postcentral gyrus and the right hippocampus, thalamus, and pallidum (*P* < 0.05, Bonferroni corrected; Fig. 3A). Lower volumes of some brain regions were associated in a graded way with lower gestational age toward extremely preterm (Additional file 1: Fig. S3). Indeed, total brain volume at adolescence was positively correlated with gestational age (*d* = 0.12, 95% CI: 0.98 to 1.03; Fig. S4). Specifically, compared to 40-week term peers, adolescents born at 36 weeks and 37–39 weeks showed the fewest brain regions with lower volume, and these brain regions involved the inferior frontal gyrus pars triangularis (BA45) and the postcentral gyrus (*P* < 0.05, Bonferroni corrected; Fig. 3B). The adolescents born at 34–35 weeks had more brain regions with lower volumes, and these were located mainly in parieto-temporal areas (*P* < 0.05, Bonferroni corrected; Fig. 3B). For adolescents born at ≤ 33 weeks, more brain regions had lower volumes, including also other parieto-temporal regions, with, in addition, frontal regions, the fusiform gyrus, cingulate cortex, insula, postcentral gyrus and the right hippocampus, thalamus, and pallidum (*P* < 0.05, Bonferroni corrected; Fig. 3B). The details of these paired comparisons are provided in Table S5. Further, the group differences related to gestational age remained similar when adolescents born with extreme birth weights (that is, large or small for gestational age) were excluded (Table S8) or when adolescents with a cesarean birth were excluded (Table S9). In addition, the group differences were not related to family income for any brain regions (interaction *P* > 0.05, Bonferroni corrected; Table S10).

### Brain volume mediates the association between gestational age and cognitive function

**Fig. 4:**
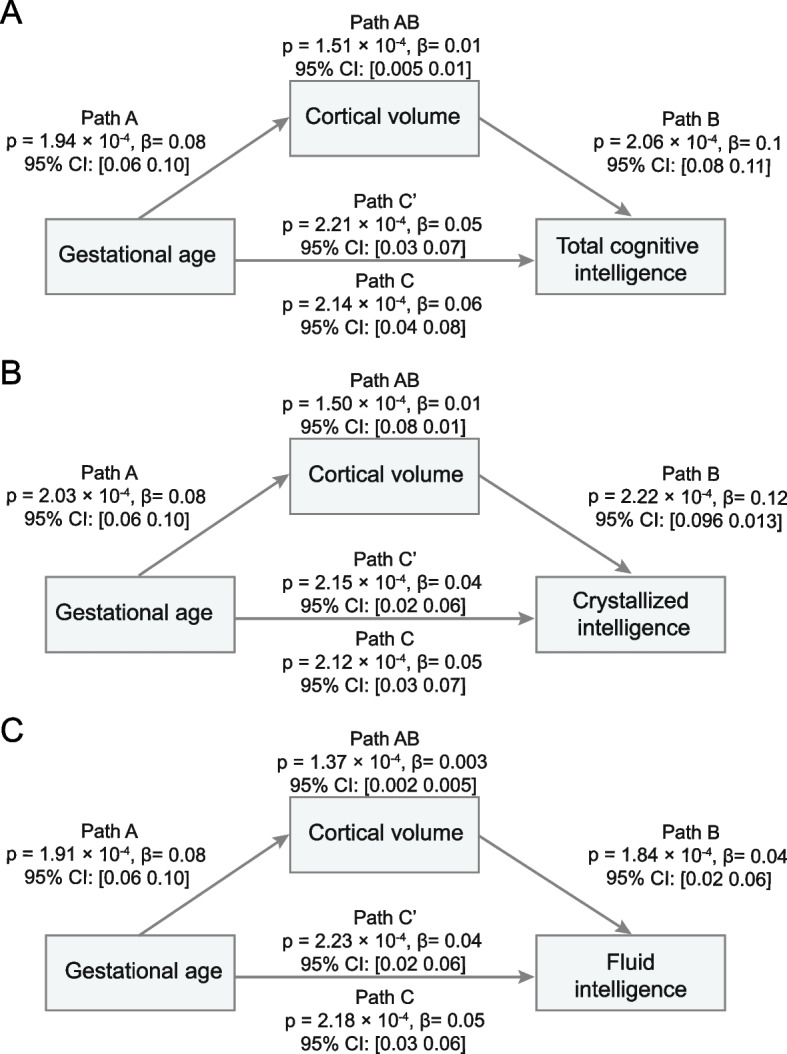
Cortical volume mediates the association between gestational age and cognitive assessments in 11,203 adolescents at baseline. The standardized mediations by cortical volume were significant from the gestational age on total intelligence (**A** *β* = 0.01, *P* = 1.51 × 10^−4^), crystallized intelligence (**B** *β* = 0.01, *P* = 1.50 × 10^−4^), and fluid intelligence (**C** *β* = 0.003, *P* = 1.37 × 10^−4^). Path A: the effect of the independent variable (gestational age) on the mediator (cortical volume); Path B: the effect of the mediator (cortical volume) on the dependent variable (cognitive intelligence); Path C: the regression coefficient (*β* value) representing the total effect of the independent variable (the gestational age) on the dependent variable (cognitive intelligence) when the mediator (cortical volume) was not taken into account; Path C’: the direct effect of the gestational age on the cognitive intelligence when controlling for the cortical volume. The regression coefficient in Path C’ shows some reduction in contrast to that in Path C. Path AB: the indirect effect of gestational age on cognitive intelligence through cortical volume can then be quantified as the product of Path A multiply by Path B. CI: confidence interval. The cortical volume used as mediator was the averaged value across regions showing significant group difference of gestational age at baseline

Mediation analysis showed that the indirect effect of the gestational age on the cognition total intelligence score was significantly mediated by the mean cortical volume of the significant brain regions shown in Fig. 3A (Path AB, 13.8% of the total effect size measured by the variance explained (VE), *β* = 0.008, 95% CI: 0.005 to 0.01, *P* = 1.5 × 10^−4^, Fig. 4A). Similar mediation effects were also found for the crystallized and fluid intelligence scores (Path AB, VE = 20%, *β* = 0.01, 95% CI: 0.007 to 0.012, *P* = 1.5 × 10^−4^, Fig. 4B; Path AB, VE = 6.67%, *β* = 0.003, 95% CI: 0.002 to 0.005, *P* = 1.4 × 10^−4^, Fig. 4C). The mediation results for the volume of specific brain region are shown in Table S6.

### Longitudinal changes of brain volume from 9–10 to 11–12 years

**Fig. 5:**
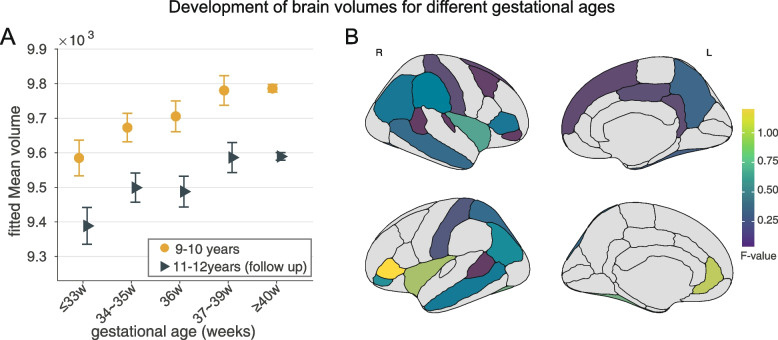
Cortical volume changes from 9–10 years to 11–12 years in 5296 adolescents. **A** Averaged brain volume in regions shown in Fig. 3A presented no significant group-by-time interaction. **B** No significant interactions of time on group differences for each region shown in Fig. 3A. The color represents the *F* value

**Fig. 6:**
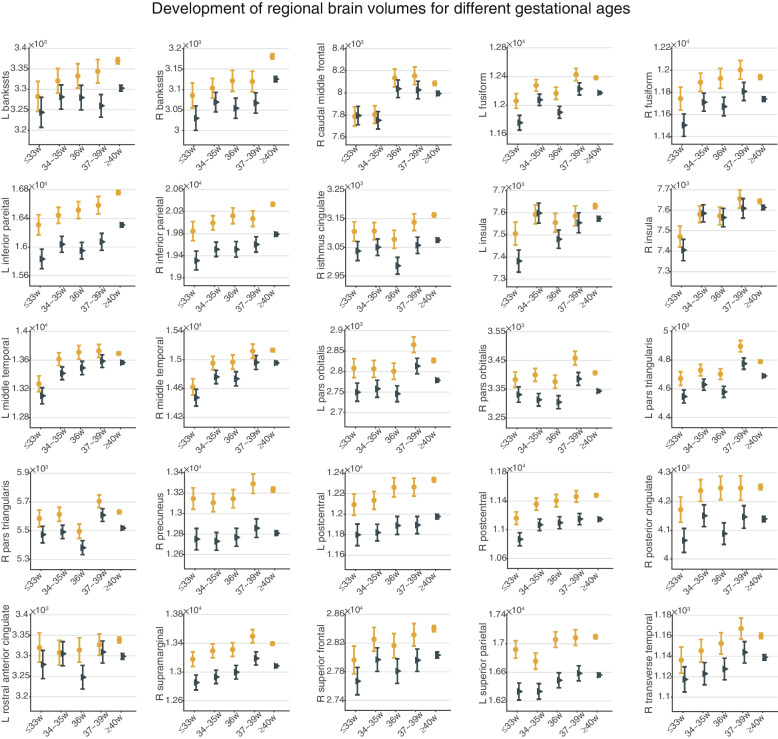
Cortical volume for each brain region changes from 9–10 years to 11–12 years in 5296 adolescents. Dot plot for regions showing no significant group by time interaction within the two-year follow-up period. banksstsrh, banks of superior temporal sulcus; L, Left; R, Right

For the brain regions showing significant between-group volume differences at baseline (as shown in Fig. 3A), the main effects remained at the follow-up, with most of those with lower gestational age still showing lower brain volumes at the follow-up, despite an overall trend to lower brain volume from baseline to follow-up (Fig. 5A and Fig. 6). Further, no significant group-by-time interactions were found for the volume of each brain region or the total brain volume (Fig. 5, Fig. 6 and Table S7), and the same findings for subcortical/non-neocortical brain regions (Fig. S5 and Table S7).

### External validation using the Danish nationwide cohort study

Analysis of data from the Danish nationwide cohort [26] showed that adolescents’ lower gestational age was associated with a lower proportion of adolescents taking the final examination (Additional file 1: Fig. S6) and lower scores for all educational domains, including problem-solving abilities and skills in Mathematics, and oral skill, reading comprehension, and spelling in Danish (Table 2, *N* = 618,070). For every week of a longer gestational duration, the problem-solving abilities, mathematical skills, oral, reading comprehension, and spelling scores go up by 0.5%, 0.6%, 0.2%, 0.5%, and 0.3%, respectively. For example, in Mathematics, we observed a positive association between GA and standardized problem-solving abilities (*β* = 0.009; 95% CI, 0.007 to 0.011). Compared to children born after 40 weeks, significantly lower scores for problem-solving abilities were observed for adolescents born less than 33 weeks (*β* =  − 0.141; 95% CI, − 0.166 to − 0.117), 34–35 weeks (*β* =  − 0.039; 95 CI%, − 0.060 to − 0.019), 36 weeks (*β* =  − 0.025; 95% CI, − 0.044 to − 0.007), and 37–39 weeks (*β* = 0.012; 95% CI, − 0.017 to − 0.006), respectively. Similar patterns were also observed in other academic domains, including mathematical skills, oral, reading comprehension, and spelling scores (as shown in Table 2 and Additional file 1: Results).

**Table 2:** Difference in standardized mean examination scores according to gestational weeks from the Danish cohort study

	Standardized mean grade difference (regression coefficient (95% CI)) ^a^				
	Mathematics 1	Mathematics 2	Danish 1	Danish 2	Danish 3
	Solving abilities	Mathematical skills	Oral	Reading comprehension	Spelling
Preterm birth					
≥ 37	REF	REF	REF	REF	REF
< 37	− 0.054 (− 0.066 to − 0.042)	− 0.077 (− 0.089 to − 0.065)	− 0.020 (− 0.032 to − 0.008)	− 0.066 (− 0.078 to − 0.054)	− 0.036 (− 0.048 to − 0.024)
Gestational weeks					
Difference ^b^	0.009 (0.007 to 0.011)	0.013 (0.011 to 0.014)	0.004 (0.002 to 0.005)	0.010 (0.008 to 0.011)	0.006 (0.004 to 0.007)
≥ 40 weeks	REF	REF	REF	REF	REF
≤ 33	− 0.141 (− 0.166 to − 0.117)	− 0.179 (− 0.203 to − 0.155)	− 0.037 (− 0.061 to − 0.012)	− 0.118 (− 0.142 to − 0.094)	− 0.051 (− 0.074 to − 0.027)
34 ~ 35	− 0.039 (− 0.060 to − 0.019)	− 0.067 (− 0.088 to − 0.047)	− 0.019 (− 0.040 to 0.001)	− 0.070 (− 0.090 to − 0.050)	− 0.037 (− 0.058 to − 0.017)
36	− 0.025 (− 0.044 to − 0.007)	− 0.040 (− 0.059 to − 0.021)	− 0.015 (− 0.034 to 0.003)	− 0.043 (− 0.062 to − 0.025)	− 0.033 (− 0.052 to − 0.015)
37 ~ 39	− 0.012 (− 0.017 to − 0.006)	− 0.017 (− 0.022 to − 0.012)	− 0.005 (− 0.011 to − 0.000)	− 0.011 (− 0.016 to − 0.006)	− 0.008 (− 0.013 to − 0.003)
*p* for trend	< 0.001	< 0.001	0.003	< 0.001	< 0.001

^a^Adjusted for sex, calendar year, parity, maternal age, paternal age, maternal education, maternal origin, maternal cohabitation at birth, maternal history of psychiatric disorders before the childbirth, and paternal history of mental disorders before the childbirth; CI, confidence interval; REF, referenced gestational week

^b^In the results of this regression model, the effect estimates represent the difference in standard deviation per 1-week longer gestational duration

^c^The effect estimates represent their differences when compared to ≥ 40-week gestation

## Discussion

This large population-based cohort study describes the relationships between gestational age and in adolescence the volume of different brain regions, cognition, and educational performance. It was found that shorter gestational age is associated in early adolescence with lower cortical volume primarily in fronto-parieto-temporal areas and lower multidimensional cognitive functions (Age, sex, body mass index, family income, parental education year, race, puberty score, maternal age at delivery, and prenatal exposure to any kind of substance before or after maternal knowledge of pregnancy were regressed out in all analyses.) Moreover, it was shown that the association between younger gestational age and lower cognitive function in early adolescence was partly mediated by the lower cortical volumes that were found. The longitudinal analysis showed that there was a decrease in the volumes of brain regions from baseline until the 2-year follow-up, but the main effects remained, with those of lower gestational age still showing lower brain volumes at the 2-year follow-up at 11–12 years. The differences of cognition for different gestational ages were in the order of 8% (Fig. 2), and the differences of brain volume in the order of 2% (Fig. 5). Analysis of data from the Danish nationwide cohort [26] provided validation of the results in that lower gestational age was associated in middle adolescents with lower scores for all educational domains (*n* = 618,070, all *P* < 0.003).

The findings extend previous studies of lower cognitive function (most of them are IQs) or poorer educational performance in very preterm or moderate preterm children [52–56], by providing evidence for more refined categories of gestational age using a large sample size (*n* = 11,878 from the ABCD cohort) together with cross-validation (*n* = 618,070 Denmark cohort), and also multi-dimensional measures of cognition, and school achievements. There were significantly lower school achievements even in the 37–39 week group compared to the full term of 40-week group that lasted until middle adolescence. This suggests that shorter gestational duration is associated with lower educational performance that is still present until at least middle adolescence.

The neuroimaging findings extend previous studies by showing that each preterm gestational group has a lower brain volume at adolescence and that lower volume is closely related to lower gestational age (Fig. 3) [57]. The graded smaller brain volumes described here for earlier gestational ages (Fig. 3B) are consistent with a recent study that treated gestational age as a continuous variable and demonstrated a positive correlation with total brain volume at 10 years [22]. Indeed, we found that total brain volume at adolescence is linearly positively correlated with gestational age (*d* = 0.12, 95% CI: 0.98 to1.03, *P* = 5.6 × 10^−11^). Here, we extend this correlation and relate lower brain volume to specific gestational age groups. We found that adolescents born at less than 36 weeks of gestation showed a wide range of lower brain volumes and that this range decreased sharply after 36 weeks, suggesting that 36 weeks may be a key point. This graded pattern was confirmed in our cognitive findings. Consistent with previous studies that frequently reported alterations in cortical gyrification, cortical thickness, surface area, and structural covariance networks in preterm youths or adults [17, 18, 21], we also found that adolescents born at less than 36 weeks presented with lower volume in temporal lobe regions. These findings suggest that children born during the third trimester may have structural differences in the temporal lobe region, one of the latest brain regions to mature [58, 59], where synaptogenesis and gyrification begin and progress rapidly throughout the third trimester [60–62]. In addition, the lower volumes in the prefrontal and parietal areas were predominantly present in adolescents born before 33 weeks’ gestation and were no longer significant in adolescents born after 33 weeks’ gestation. Our findings in this large cohort thus suggest that different gestational ages may be associated with differences in different brain regions. A previous study also found that the volume in the prefrontal region was lower in a cohort of 29 preterm (< 30 weeks) children at 12 years [63]. Further, lower brain volumes in preterm adolescents may reflect less brain development in utero and/or underdevelopment during postnatal maturation [58].

The brain regions with lower volume associated with lower gestational age are illustrated in Figs. 3 and 5 and listed in Table S5. The lateral temporal lobe regions include cortex in the banks of the superior temporal sulcus that is involved in representing socially relevant visual stimuli such as face expression and movements [64–67] and in the semantic representations involved in language [68]. The inferior prefrontal regions including Broca’s area and the temporal-parietal regions are also implicated in language [68]. The postcentral gyrus and connected insula and inferior and medial parietal cortex regions are involved in somatosensory processing and representing actions in space [69–71]. The hippocampal regions are involved in memory [72–74]. The orbitofrontal and anterior cingulate cortex are involved in emotion [75, 76].

A previously unanswered critical question is the underlying mechanism in the perinatal period that affects the neurodevelopmental risks. The present study advances previous research by identifying brain regions the volumes of which significantly mediate the association between GA and cognitive function. In addition, longitudinal analysis revealed that the lower cortical volumes found in preterm groups are consistent with typical maturational processes around adolescence [77–79]. For instance, one study showed a decline in brain volumes from 8 to12 years [8] and another study using a relatively larger sample size also found volumetric decrease from 8 to 23 years [3]. Various biological mechanisms are proposed to underlie lower cortical volumes, including slowed cell growth, decreased dendritic arbor size, and elimination of synapses [61]. Another key finding here is that even by age 11–12, the lower GA groups still had lower cortical volumes. The trajectories described here are consistent with those found in recent studies on cortical volume in very preterm adolescents (< 30 weeks’ gestation) or of very low birthweight (< 1250 g) children [19]. A similar developmental pattern was also found for cortical thickness and surface area in preterm children and adolescents (< 37 gestational weeks) with very low birth weight [20]. All these evidences may suggest a “lack of catch-up” of brain structural growth in the preterm groups, which potentially reflects an altered brain developmental route following prenatal maturational differences, and supports the view that brain development in the postnatal years after premature birth may not compensate [80].

### Strengths and limitations

Several strengths of the research are described here. First, this is a large-scale (*n* = 11,878), retrospective cohort study characterized by refined categories of gestational weeks and comprehensive covariate data integrating with cognitive assessments. The longitudinal design for the study enabled us to track the growth trajectories two years later in brain regions with their volumes significantly associated with gestational age and cognition. Second, in addition to regressing out a variety of prenatal and postnatal covariates related to family variables, pregnancy, and adolescence (see the “ Methods” section), we also considered variables closely related to gestational age (i.e., birth weight and cesarean delivery), providing a reasonable and rigorous model for exploring the association between gestational age and neural and cognitive development until adolescence. Third, the nationwide setting of this database and further validation with a much larger sample size from the Danish cohort (*n* = 618,070) enabled the association between gestational age and adolescent development to be extended from research tests to educational performance.

Several limitations need to be considered. First, causal relationships cannot be inferred from our retrospective cohort design, which is primarily an association study. The exact biological mechanism underlying the association between gestational age and postnatal maturation needs to be further explored. Second, recall bias should be considered because detailed gestational age information during pregnancy was based on questionnaires in this database. For example, the current ABCD database defined term birth as having 40 weeks of gestation which may include a small portion of participants born at greater than 40 weeks. Further exploration is needed to examine a wider range of gestational age from preterm to post-term gestation (i.e., > 40 weeks). Third, the current categories of gestational age are not based on each gestational week. Certain gestational weeks were grouped together due to smaller number of participants born for some gestational ages. Therefore, it limited our understanding of how the brain develops in early adolescence with week-to-week gestations. Fourth, complete data for 2-year follow-up cognitive abilities are not available, which makes exploration of the longitudinal development of cognitive performance not possible. But at least, for the only available data of crystallized intelligence scores, we found lower cognitive performance still present in preterm groups at 11–12 years, suggesting that cognitive differences may also be long-lasting. Fifth, although we tried to control for as many relevant variables as possible, we could not control for every possible variable. In future studies, if available, more variables related to prematurity and adolescents’ cognitive functioning could also be considered, such as pre- and postnatal nutrition and access to health care. Sixth, the effect size for lower brain volume was smaller in the near-term preterm group than in the earlier preterm groups, so for preterm adolescents born close to the full-term week, the clinical significance may be lower. However, brain volume in all preterm groups was stably lower than that in the term group at both 9–10 years and at the 2-year later follow-up. Lastly, the participants of this retrospective cohort study were extracted from a database from the United States, and therefore, the generalization to other countries and areas requires further validation.

## Conclusions

In conclusion, this study provides new large-scale evidence about how lower gestational ages are associated at adolescence with lower volumes of some brain regions and with lower cognition. This relation between gestational age and subsequent school achievements was further demonstrated in a very large Danish cohort (*n* = 618,070). The study indicates the importance of early screening and close follow-up for neurocognitive and behavioral development for children and adolescents born with gestational ages that are even a little lower than full term.

## Supplementary Information

**Additional file 1: Table S1.** Demographic characteristics of adolescents atninth-grade born with different gestational weeks from the Danish cohort study.**Table S2.** Group difference for gestational age and cognitive measures at 9-10 years. **Table S3.** Validation of group difference for gestational age andcognitive measures at 9-10 years. **Table S4.** Group difference for gestational age and cognitive measures at 11-12years. **Table S5.** Groupdifference for the association between gestational age and brain volume in adolescents at 9-10 years. **Table S6.**Mediation result for mediation by each volumetric region of the association between gestational age and cognitive measures in adolescents at 9-10 years. **Table S7.** Longitudinal group-by-time interaction for brain volume in adolescents from 9-10 years to 11-12 years. **Table S8.** Group difference for theassociation between gestational age and brain volume when excluding adolescentswith extreme birth weight at 9-10 years. **Table S9.** Group difference for the association between gestationalage and brain volume at 9-10 years when excluding adolescents with Caesareian birth. **Table S10.** Group-by-income interaction for brain volumes in adolescents at 9-10 years. **Fig. S1.** The distribution of gestational weeks at both baseline and 2-years follow-up neuroimaging analyses.**Fig. S2.** Differences of cognitive performance between any two gestational weeks. **Fig. S3.** Brain regions with lower volumes for different gestational age. **Fig. S4.** Positive correlation between gestational age and whole brain volumes at baseline. **Fig. S5.**Subcortical/non-neocortical volume change from 9-10 years to 11-12 years. **Fig. S6.** Proportion of adolescents taking the final examination according to gestational weeks from the Danish cohort study.

## Data Availability

Neuroimaging and cognitive data from the ABCD dataset are available from. https://nda.nih.gov/abcd with the approval of the ABCD consortium. The datasets for Danish cohort are not open access.

## Abbreviations

ABCD: Adolescent brain cognitive development
ASEG: Automatic subcortical segmentation
GA: Gestational age
LGA: Large for gestational age
LMM: Linear mixed model
MRI: Magnetic resonance imaging
SGA: Small for gestational age
