# Supplementary material for: Lower gestational age is associated with lower cortical volume and cognitive and educational performance in adolescence
Source: BMC Med. 2022 Nov 3;20:424. doi: 10.1186/s12916-022-02627-3 (PMC9635194; doi:10.1186/s12916-022-02627-3)
Supplement: Supplementary file 1 — Additional file 1: Table S1. Demographic characteristics of adolescents atninth-grade born with different gestational weeks from the Danish cohort study.Table S2. Group difference for gestational age and cognitive measures at 9-10 years. Table S3. Validation of group difference for gestational age andcognitive measures at 9-10 years. Table S4. Group difference for gestational age and cognitive measures at 11-12years. Table S5. Groupdifference for the association between gestational age and brain volume in adolescents at 9-10 years. Table S6.Mediation result for mediation by each volumetric region of the association between gestational age and cognitive measures in adolescents at 9-10 years. Table S7. Longitudinal group-by-time interaction for brain volume in adolescents from 9-10 years to 11-12 years. Table S8. Group difference for theassociation between gestational age and brain volume when excluding adolescentswith extreme birth weight at 9-10 years. Table S9. Group difference for the association between gestationalage and brain volume at 9-10 years when excluding adolescents with Caesareian birth. Table S10. Group-by-income interaction for brain volumes in adolescents at 9-10 years. Fig. S1. The distribution of gestational weeks at both baseline and 2-years follow-up neuroimaging analyses.Fig. S2. Differences of cognitive performance between any two gestational weeks. Fig. S3. Brain regions with lower volumes for different gestational age. Fig. S4. Positive correlation between gestational age and whole brain volumes at baseline. Fig. S5.Subcortical/non-neocortical volume change from 9-10 years to 11-12 years. Fig. S6. Proportion of adolescents taking the final examination according to gestational weeks from the Danish cohort study. [file 12916_2022_2627_MOESM1_ESM.docx]

**Supplementary Online Content**

**Lower gestational age is associated with lower cortical volume and cognitive and educational performance in adolescence**

Qing Ma, PhD^1,2#^; Hui Wang, PhD^3#^; Edmund T. Rolls, DPhil, DSc^1,5,6#^; Shitong Xiang, MS^1,2^; Jiong Li, PhD^7^; Yuzhu Li, MS^1,2^; Qiongjie Zhou, MD, PhD^8,9^; Wei Cheng, PhD^1,2,4,10*^; Fei Li, MD^3*^

1. Department of Neurology, Huashan Hospital, Institute of Science and Technology for Brain-Inspired Intelligence, State Key Laboratory of Medical Neurobiology and MOE Frontiers Center for Brain Science, Fudan University, Shanghai, 200433, China

2. Key Laboratory of Computational Neuroscience and Brain-Inspired Intelligence, Fudan University, Ministry of Education, Shanghai, 200433, China

3. Department of Developmental and Behavioral Pediatric & Child Primary Care/MOE-Shanghai Key Laboratory of Children’s Environmental Health, Xin Hua Hospital Affiliated to Shanghai Jiao Tong University School of Medicine, Shanghai, 200082, China

4. Fudan ISTBI—ZJNU Algorithm Centre for Brain-inspired Intelligence, Zhejiang Normal University, Jinhua, 321004, China

5. Department of Computer Science, University of Warwick, Coventry CV4 7AL, UK

6. Oxford Centre for Computational Neuroscience, Oxford, UK

7. Department of Clinical Medicine, Aarhus University, Aarhus, 8000, Denmark

8. Obstetrics and Gynecology Hospital of Fudan University, Shanghai, 200011, China

9. Shanghai Key Laboratory of Female Reproductive Endocrine-Related Diseases, Shanghai, 200011, China

10. Shanghai Medical College and Zhongshan Hospital Immunotherapy Technology Transfer Center, Shanghai, 200032, China

# These authors contributed equally to this work.

*** Corresponding author**

Wei Cheng, Department of Neurology, Huashan Hospital, Institute of Science and Technology for Brain-Inspired Intelligence, State Key Laboratory of Medical Neurobiology and MOE Frontiers Center for Brain Science, Fudan University, Shanghai 200433, China.

E-mail address: wcheng@fudan.edu.cn

Fei Li, Department of Developmental and Behavioral Pediatric & Child Primary Care/MOE-Shanghai Key Laboratory of Children’s Environmental Health, Xin Hua Hospital Affiliated to Shanghai Jiao Tong University School of Medicine, Shanghai, 200082, China.

E-mail address: [lifei5861_cn@163.com](mailto:lifei5861_cn@163.com)

**Methods**

Neuroimaging data preprocessing

Confounding factors

External validation analysis using the Danish nationwide cohort study

**Results**

Participant characteristics

Results for Confounding factors

External validation results using the Danish nationwide cohort study

**Table S1.** Demographic characteristics of adolescents at ninth-grade born with different gestational weeks from the Danish cohort study

**Table S2.** Group difference for gestational age and cognitive measures at 9-10 years

**Table S3.** Validation of group difference for gestational age and cognitive measures at 9-10 years

**Table S4.** Group difference for gestational age and cognitive measures at 11-12 years

**Table S5.** Group difference for the association between gestational age and brain volume in adolescents at 9-10 years

**Table S6.** Mediation result for mediation by each volumetric region of the association between gestational age and cognitive measures in adolescents at 9-10 years

**Table S7.** Longitudinal group-by-time interaction for brain volume in adolescents from 9-10 years to 11-12 years

**Table S8.** Group difference for the association between gestational age and brain volume when excluding adolescents with extreme birth weight at 9-10 years

**Table S9.** Group difference for the association between gestational age and brain volume at 9-10 years when excluding adolescents with Caesarian birth

**Table S10.** Group-by-income interaction for brain volumes in adolescents at 9-10 years

**Figure S1.** The distribution of gestational weeks at both baseline and 2-years follow-up neuroimaging analyses.

**Figure S2.** Differences of cognitive performance between any two gestational weeks

**Figure S3.** Brain regions with lower volumes for different gestational age

**Figure S4.** Positive correlation between gestational age and whole brain volumes at baseline

**Figure S5.** Subcortical/non-neocortical volume change from 9-10 years to 11-12 years

**Figure S6.** Proportion of adolescents taking the final examination according to gestational weeks from the Danish cohort study.

**Methods**

**Participants**

Participants were from the most recent release 3.0 of the ABCD study (<https://abcdstudy.org/scientists/data-sharing/>), which is an ongoing multi-site longitudinal investigation of Adolescent Brain Cognitive Development in the United States, and they and their parents or caregivers completed a set of visits consisting of clinical interviews, surveys, neurocognitive tests and neuroimaging. The visits for the population are ongoing every year for behavioral and cognitive assessments and every two years for neuroimaging scanning. Parents provided written informed consent and each adolescent provided written assent. More details of the subjects and the collection are provided at the ABCD website (<https://abcdstudy.org/scientists/protocols/>) and elsewhere[27, 29].

**Neuroimaging data preprocessing**

Preprocessed structural imaging data (T1 and T2) using the ABCD pipeline were obtained, with all the data preprocessing procedures performed by the ABCD team as described in their neuroimaging processing paper[33]. The data preprocessing included the following procedures: 1) T1w and T2w structural images were corrected for gradient nonlinearity distortions; 2) T2w images were registered to T1w images; 3) intensity normalization and B1 inhomogeneity correction; 4) images were rigidly registered and resampled into alignment with a custom, in-house atlas created by the ABCD data preprocessing team for participants of this age; 5) FreeSurfer (version: v5.3.0) was used for cortical surface reconstruction and subcortical segmentation which included skull-stripping, white matter segmentation, initial mesh creation, correction of topological defects, generation of optimal white and pial surfaces, and nonlinear registration to a spherical surface-based atlas based on the alignment of sulcal/gyral patterns; 6) images were registered to a spherical atlas based on surface-based nonlinear registration, and the cerebral cortex was parcellated into 34 regions per hemisphere; 7) subcortical structures were labeled using an automated, atlas-based, volumetric segmentation procedure. The quality control of the processed images was done by the trained staff from the ABCD team, combining both manual and automated review for the accuracy of cortical surface reconstruction. The final use of neuroimages in the current study was chosen using variable “abcd_imgincl01” from the ABCD data dictionary (https://nda.nih.gov/data_structure.html?short_name=abcd_imgincl01), with 1 indicating inclusion and 0 exclusion.

**External validation analysis using the Danish nationwide cohort study**

In Denmark, all live births have a unique personal identification number that permits an accurate linkage of individual-level data[26, 46-49]. We identified all singleton live born infants (N=643,878) from January 1^st^, 1991 to December 31^st^, 2000 in the Danish Medical Birth Registry (DMBR). We excluded infants who had missing data on gestational age (N=27,725), without information on sex (N=86), and/or with chromosomal abnormalities (N=2,118), dead (N=4,207) or emigrated before 18 (N=11,672), and the final analysis included 618,070 in Denmark.

Information on gestational age was obtained from the DMBR[26]. Gestational age in completed weeks of gestation was first categorized into two groups: <37 (preterm birth) and ≥37 (term birth) gestational weeks, then categorized into the following groups: less than 33, 34-35, 36, 37-39, and ≥40 (full term) gestational weeks.

The first measure of academic achievement was taking the exit exam at the ninth-grade, which was obtained from the Danish Education Register[47]. The exit exam marks the end of compulsory schooling in Denmark and assesses the individual’s eligibility for upper secondary education at the age of 15-16 years. The second measure was the examination grades in Danish and Mathematics. These two subjects were chosen because they were compulsory and all children throughout the Demark took identical tests in Danish and Mathematics. The Danish test evaluates a student’s ability within 3 profile areas including oral, reading comprehension, and spelling. In Mathematics, there are problem-solving abilities and mathematic skills. These tests are computer-based, adaptive, and objective, which cannot be influenced by teachers.

The Danish authorities used a seven-point grading system[47]. This system was adopted only since 2007. Before 2007, a ten-point grading system was used. In this study, the grades from the old grading scale before 2007 were converted to seven-point grades using the conversion table provided by the Danish Ministry of Education. We standardized the grades as z scores by subtracting the mean and dividing by the standard deviation (SD).

The proportions of individuals who took the exit examination at the end of the ninth grade were calculated across gestational weeks. In addition, we used a multivariable linear regression model to estimate the differences in school grades across the gestational weeks, presenting the regression coefficients with 95% confidence interval (CI) to express the mean differences. Based on previous research, the following variables were considered as potential confounders and were regressed out: sex of the adolescent (male, female), calendar period of birth (a 2-year interval during 1991-2000), parity (1, 2, ≥3), maternal age at birth (≤25, 26-30, 31-35, ≥36 years), paternal age at birth (≤25, 26-30, 31-35, ≥36 years), maternal country of origin (Denmark, other countries), maternal education level (0-9, 10-14, ≥15 years), maternal cohabitation status at birth (yes, no), maternal psychiatric disorder before the childbirth (yes, no), and maternal smoking during pregnancy (yes, no).

**Results**

**Participant characteristics**

Several characteristics were observed showing significant group differences at baseline, including adolescents’ age (partial eta-square [η^2^_p_]=0.005, 95% CI: 0.002 to 0.007), body mass index (BMI, η^2^_p_=0.002, 95% CI: 0.0005 to 0.0037), racial type (Cramer’s V=0.04, 95% CI:0.03 to 0.05) and puberty score (η^2^_p_=0.001, 95% CI: 0.00007 to 0.002), as well as other measures from the family, such as maternal age at birth (η^2^_p_=0.002, 95% CI: 0.0007 to 0.004), parental total income (categorized as low-level (<$50,000), middle-level ($50,000-100,000) and high-level (>$100,000); V=0.05, 95% CI: 0.04 to 0.06) and parental education level (η^2^_p_=0.003, 95% CI: 0.001 to 0.006). No significant group differences were found for gender, or for prenatal exposure of any substances before and after knowing of pregnancy.

**Results for confounding factors**

To control the possible confounding effect of birth weight, a total of 282 adolescents of large for gestational age and 950 of small for gestational age were excluded from the baseline population cohort. We detected similar group-differences as before the participants were excluded **(Table S6)**.

The determination of Caesarian birth was based on parental answer to the item of “Was your child born by Caesarian section?” (devhx_13_13_p). Of the total population, 146 parents reported “don’t know” or the answer was missing, and 4,443 adolescents were born by Caesarian delivery, with 323, 351, 329, 277, 3,163 adolescents born with a gestational age less than 33 weeks, 34~35 weeks, 36 weeks, 37~39 weeks (early term), and ≥40 gestational weeks (full term), respectively. For the spontaneous born adolescents, there were a total of 7,258 participants with 130, 201, 260, 300, 6367 born in gestation of less than 33 weeks, 34~35 weeks, 36 weeks, 37~39 weeks, and ≥40 weeks, respectively. We detected similar group-differences in the spontaneous born adolescents as the main findings **(Table S7).**

**External validation results using the Danish nationwide cohort study**

Overall, adolescents born with lower numbers of gestational weeks were more likely to be boys and had higher percentages of nulliparous mothers. Mothers of offspring with low numbers for the gestational weeks were more likely to be older, with lower education level, of non-Danish origin, or had more comorbid psychiatric disorders. Of the total study population, 4.53% of the births were born before 37 weeks of gestation, and 1.15%, 1.54%, 1.84%, 36.46%, and 59.02% of the adolescents were born with a gestational age less than 33 weeks, 34~35 weeks, 36 weeks, 37~39 weeks (early term), and ≥40 gestational weeks (full term), respectively.

In all, 91.8% of the individuals took the final examination. Of the adolescents born before 37 gestational weeks, 88.2% (95% CI, 87.8% to 88.6%) took the final examination compared with 92.0% (95% CI, 91.9% to 92.0%) of those born at term. The percentage of adolescents who took the exam decreased stepwise with decreasing gestational age, from 92.2% (95% CI, 92.2% to 92.3%) at more than 40 gestational weeks to 85.2% (95% CI, 84.3% to 86.0%) at less than 33 gestational weeks. (**Figure S2**)

In the linear regression analysis, adolescents had lower scores for all academic domains with the lower gestational weeks. For the problem-solving abilities in Mathematics, the results are described in the main text. For Mathematic skills, the adjusted mean differences were -0.179 (-0.203 to -0.155), -0.067 (-0.088 to -0.047), -0.040 (-0.059 to -0.021), and -0.017 (-0.022 to -0.012) for adolescents born less than 33 gestational weeks, 34~35 gestational weeks, 36 gestational weeks, and 37~39 gestational weeks, respectively, compared to adolescents born after 40 gestational weeks. Similar patterns were observed in Oral skill in Danish for five groups, with adjusted mean difference -0.037 (-0.061 to -0.012), -0.019 (-0.040 to 0.001), -0.015 (-0.034 to 0.003) and -0.005 (-0.011 to -0.000) respectively. For reading comprehension in Danish, the adjusted mean differences were -0.118 (-0.142 to -0.094), -0.070 (-0.090 to -0.050), -0.043 (-0.062 to -0.025), and -0.011 (-0.016 to -0.006). The adjusted mean difference for spelling in Danish were -0.051 (-0.074 to -0.027), -0.037 (-0.058 to -0.017), -0.033 (-0.052 to -0.015) and -0.008 (-0.013 to -0.003). All of the information can be seen in **Table 2**.

**Table S1.** Demographic characteristics of adolescents at ninth-grade born with different gestational weeks from the Danish cohort study

| Characteristics |  | Gestational age | | | | | | | |  |  |
| --- | --- | --- | --- | --- | --- | --- | --- | --- | --- | --- | --- |
|  |  | < 37 week | ≥ 37 week | ≤33 week | 34~35 week | 36 week | | 37~39 week | ≥40 week | |  |
| Sex, N (%) | Boys | 15 439 (55.2) | 301 793 (51.1) | 3906 (55.0) | 5295 (55.8) | 6238 (54.8) | | 117 184 (52.0) | 184 609 (50.6) | |  |
|  | Girls | 12 534 (44.8) | 288 304 (48.9) | 3193 (45.0) | 4202 (44.2) | 5139 (45.2) | | 108 146 (48.0) | 180 158 (49.4) | |  |
| Year of birth | 1991-1992 | 5577 (19.9) | 118 618 (20.1) | 1421 (20.0) | 1863 (19.6) | 2293 (20.2) | | 44 734 (19.8) | 73 884 (20.3) | |  |
|  | 1993-1994 | 5463 (19.5) | 122 875 (20.8) | 1392 (19.6) | 1830 (19.3) | 2241 (19.7) | | 45 241 (20.1) | 77 634 (21.3) | |  |
|  | 1995-1996 | 5836 (20.9) | 122 011 (20.7) | 1448 (20.4) | 2004 (21.1) | 2384 (20.9) | | 46 785 (20.8) | 75 226 (20.6) | |  |
|  | 1997-1998 | 5265 (18.8) | 111 260 (18.9) | 1321 (18.6) | 1838 (19.3) | 2106 (18.5) | | 42 323 (18.8) | 68 937 (18.9) | |  |
|  | 1999-2000 | 5832 (20.9) | 115 333 (19.5) | 1517 (21.4) | 1962 (20.7) | 2353 (20.7) | | 46 247 (20.5) | 69 086 (18.9) | |  |
| Parity | 1 | 14 627 (52.3) | 255 208 (43.2) | 3717 (52.4) | 5133 (54.1) | 5777 (50.8) | | 94 724 (42.0) | 160 484 (44.0) | |  |
|  | 2 | 8335 (29.8) | 224 084 (38.0) | 2057 (29.0) | 2749 (28.9) | 3529 (31.0) | | 85 606 (38.0) | 138 478 (38.0) | |  |
|  | ≥ 3 | 5011 (17.9) | 110 805 (18.8) | 1325 (18.6) | 1615 (17.0) | 2071 (18.2) | | 45 000 (20.0) | 65 805 (18.0) | |  |
| Maternal age (years) | ≤ 25 | 5994 (21.4) | 107 424 (18.3) | 1491 (21.0) | 2116 (22.3) | 2387 (21.0) | | 41 877 (18.6) | 65 547 (18.0) | |  |
|  | 26-30 | 10 445 (37.3) | 232 678 (39.4) | 2496 (35.2) | 3579 (37.7) | 4370 (38.4) | | 86 661 (38.5) | 146 017 (40.0) | |  |
|  | 31-35 | 7818 (28.0) | 181 355 (30.7) | 2081 (29.3) | 2546 (26.8) | 3191 (28.1) | | 68 523 (30.4) | 112 832 (30.9) | |  |
|  | ≥ 36 | 3716 (13.3) | 68 640 (11.6) | 1031 (14.5) | 1256 (13.2) | 1429 (12.5) | | 28 269 (12.5) | 40 371 (11.1) | |  |
| Paternal age (years) | ≤ 25 | 4242 (15.4) | 74 746 (12.8) | 1076 (15.4) | 1507 (16.1) | 1659 (14.8) | | 29 129 (13.1) | 45 617 (12.6) | |  |
|  | 26-30 | 9578 (34.8) | 200 792 (34.5) | 2345 (33.6) | 3271 (35.0) | 3962 (35.3) | | 75 780 (34.1) | 125 012 (34.7) | |  |
|  | 31-35 | 7960 (28.9) | 187 764 (32.2) | 2026 (29.0) | 2662 (28.5) | 3272 (29.2) | | 70 515 (31.7) | 117 249 (32.6) | |  |
|  | ≥ 36 | 5764 (20.9) | 119 226 (20.5) | 1539 (22.0) | 1902 (20.4) | 2323 (20.7) | | 46 900 (21.0) | 72 326 (20.1) | |  |
| Maternal education (years) | 0-9 | 9128 (33.0) | 152 758 (26.1) | 2401 (34.2) | 3064 (32.6) | | 3663 (32.5) | 62 311 (27.9) | 90 447 (25.0) | | |
|  | 10-14 | 13437 (48.5) | 294 831 (50.3) | 3352 (47.7) | 4599 (48.9) | | 5486 (48.7) | 111 568 (49.9) | 183 263 (50.5) | | |
|  | ≥ 15 | 5126 (18.5) | 138 311 (23.6) | 1272 (18.1) | 1735 (18.5) | | 2119 (18.8) | 49 573 (22.2) | 88 738 (24.5) | | |
| Maternal cohabitation at birth | Yes | 14 314 (51.2) | 272 148 (46.1) | 3679 (51.8) | 4944 (52.1) | | 5 691 (50.0) | 102 713 (45.6) | 169 435 (46.5) | | |
|  | No | 13 659 (48.8) | 317 944 (53.9) | 3420 (48.2) | 4553 (47.9) | | 5 686 (50.0) | 122 615 (54.4) | 195 329 (53.5) | | |
| Maternal origin | Not Danish | 2846 (10.2) | 55 698 (9.4) | 688 (9.7) | 951 (10.0) | | 1207 (10.6) | 24 852 (11.0) | 30 846 (8.5) | | |
|  | Danish | 25 118 (89.8) | 534 289 (90.6) | 6404 (90.3) | 8546 (90.0) | | 10 168 (89.4) | 200 436 (89.0) | 333 853 (91.5) | | |
| Maternal mental disorders  before the childbirth | No | 26 477 (94.7) | 569 969 (96.6) | 6695 (94.3) | 8988 (94.6) | | 10 794 (94.9) | 216 555 (96.1) | 353 414 (96.9) | | |
|  | Yes | 1496 (5.3) | 20 128 (3.4) | 404 (5.7) | 509 (5.4) | | 583 (5.1) | 8775 (3.9) | 11 353 (3.1) | | |
| Paternal mental disorders  before the childbirth | No | 26 758 (95.7) | 570 571 (96.7) | 6777 (95.5) | 9090 (95.7) | | 10 891 (95.7) | 217 397 (96.5) | 353 174 (96.8) | | |
|  | Yes | 1215 (4.3) | 19 526 (3.3) | 322 (4.5) | 407 (4.3) | | 486 (4.3) | 7933 (3.5) | 11 593 (3.2) | | |

The values represented mean (std).

**Table S2.** Group difference for gestational age and cognitive measures at 9-10 years

| Cognition | Main Effect  F (*P*) values | Simple Effect  t (*P*) values | | | |
| --- | --- | --- | --- | --- | --- |
|  |  | ≤33 week | 34~35 week | 36 week | 37~39 week |
| nihtbx_totalcomp_fc | **16.7 (1.1×10^-13^)** | -6.91 (5.1×10^-12^) | -3.72 (2.0×10^-4^) | -3.33 (8.8×10^-4^) | -0.94 (0.35) |
| nihtbx_cryst_fc | **17.1 (5.7×10^-14^)** | -6.77 (1.5×10^-11^) | -4.62 (4.0×10^-6^) | -2.52 (0.01) | -1.39 (0.16) |
| nihtbx_fluidcomp_fc | **7.37 (6.3×10^-6^)** | -4.56 (5.1×10^-6^) | -1.68 (0.09) | -2.84 (5.0×10^-3^) | -0.04 (0.97) |
| nihtbx_reading_fc | **5.71 (1.4×10^-4^)** | -4.15 (3.3×10^-5^) | -2.64 (0.01) | -1.18 (0.24) | -0.55 (0.58) |
| nihtbx_picvocab_fc | **11.3 (4.1×10^-9^)** | -5.93 (3.1×10^-9^) | -3.15 (2.0×10^-3^) | -1.76 (0.08) | -0.84 (0.40) |
| nihtbx_list_fc | **8.67 (5.6×10^-7^)** | -5.56 (2.8×10^-8^) | -1.99 (0.05) | -1.33 (0.18) | -0.49 (0.62) |
| nihtbx_picture_fc | **3.94 (0.003)** | -3.35 (8.2×10^-4^) | -0.3 (0.77) | -1.82 (0.07) | 1.07 (0.28) |
| nihtbx_flanker_fc | 1.86 (0.11) | -2.01 (0.04) | -1.42 (0.16) | -1.45 (0.15) | -0.08 (0.94) |
| nihtbx_cardsort_fc | 1.93 (0.10) | -2.23 (0.03) | -1.39 (0.17) | -0.64 (0.53) | 0.75 (0.45) |
| nihtbx_pattern_fc | 2.49 (0.04) | -1.88 (0.06) | -0.34 (0.73) | -2.69 (0.01) | -0.33 (0.74) |

Gestational age of more than 40 weeks was taken as the reference group in the simple effect test. All of the displayed *P* values were the original ones. Bold values indicated significance with Bonferroni correction (*P*<0.05). Note: No multiple comparison correction was applied to the simple effect test because it is a post-hoc analysis following main effect detection. Abbreviations: nihtbx_totalcomp_fc: Cognition Total Composite Score Fully-Corrected T-score; nihtbx_cryst_fc: Crystallized Composite Fully-Corrected T-score; nihtbx_fluidcomp_fc: Cognition Fluid Composite Fully-Corrected T-score; nihtbx_reading_fc: NIH Toolbox Oral Reading Recognition Test Age 3+ v2.0 Fully-Corrected T-score; nihtbx_picvocab_fc: NIH Toolbox Picture Vocabulary Test Age 3+ v2.0 Fully-Corrected T-score; nihtbx_list_fc: NIH Toolbox List Sorting Working Memory Test Age 7+ v2.0 Fully-Corrected T-score; nihtbx_picture_fc: NIH Toolbox Picture Sequence Memory Test Age 8+ Form A v2.0 Fully-Corrected T-score; nihtbx_flanker_fc: NIH Toolbox Flanker Inhibitory Control and Attention Test Ages 8-11 v2.0 Fully-Corrected T-score; nihtbx_cardsort_fc: NIH Toolbox Dimensional Change Card Sort Test Ages 8-11 v2.0 Fully-Corrected T-score; nihtbx_pattern_fc: NIH Toolbox Pattern Comparison Processing Speed Test Age 7+ v2.0 Fully-Corrected T-score.

**Table S3.** Validation of group difference for gestational age and cognitive measures at 9-10 years

|  | **Main Effect**  F (*P*) values | **Simple Effect**  t (*P*) values | | | |
| --- | --- | --- | --- | --- | --- |
|  |  | ≤33 weeks | 34~35 weeks | 36 weeks | 37~39 weeks |
| Little Man Task | | | | | |
| lmt_scr_perc_correct | 8.25 (1.2×10^-6^) | -3.51 (5×10^-4^) | -3.65 (0.0003) | -2.63 (0.009) | 1.38 (0.169) |
| lmt_scr_perc_wrong | 5.89 (9.9×10^-5^) | 2.99 (0.003) | 3.22 (0.001) | 2.24 (0.025) | -0.78 (0.433) |
| lmt_scr_num_correct | 8.25 (1.2×10^-6^) | -3.51 (5×10^-4^) | -3.65 (0.0003) | -2.63 (0.009) | 1.38 (0.169) |
| lmt_scr_num_wrong | 5.89 (9.9×10^-5^) | 2.99 (0.003) | 3.22 (0.001) | 2.24 (0.025) | -0.78 (0.434) |
| Rey Auditory Verbal Learning Test | | | | | |
| pea_ravlt_sd_trial_i_tc | 6.28 (4.9×10^-5^) | -4.00 (6×10^-5^) | -1.70 (0.089) | -2.89 (0.004) | -0.10 (0.923) |
| pea_ravlt_sd_trial_ii_tc | 5.75 (1×10^-4^) | -3.73 (2×10^-4^) | -0.89 (0.373) | -2.93 (0.003) | 0.73 (0.463) |
| pea_ravlt_sd_trial_iii_tc | 5.25 (3×10^-4^) | -3.83 (1×10^-4^) | -0.96 (0.337) | -2.63 (0.009) | -0.40 (0.688) |
| pea_ravlt_sd_trial_iv_tc | 5.24 (3×10^-4^) | -3.81 (1×10^-4^) | -1.42 (0.156) | -2.29 (0.022) | 0.54 (0.593) |
| pea_ravlt_sd_trial_v_tc | 6.74 (2.1×10^-5^) | -4.74 (2.2×10^-6^) | -0.90 (0.368) | -2.05 (0.041) | 0.59 (0.553) |
| pea_ravlt_sd_listb_tc | 2.77 (0.026) | -2.41 (0.016) | -2.19 (0.029) | -1.10 (0.27) | 0.24 (0.813) |
| pea_ravlt_sd_trial_vi_tc | 7.33 (6.8×10^-6^) | -4.96 (7.0×10^-7^) | -1.21 (0.226) | -1.53 (0.126) | 1.12 (0.264) |
| pea_ravlt_ld_trial_vii_tc | 4.38 (0.002) | -3.46 (0.001) | -0.70 (0.487) | -2.00 (0.046) | 1.12 (0.264) |
| Matrix Reasoning Task | | | | | |
| pea_wiscv_trs | 2.93 (0.019) | -2.99 (0.003) | -1.74 (0.082) | -0.72 (0.471) | -0.01 (0.992) |
| pea_wiscv_tss | 3.06 (0.016) | -2.90 (0.004) | -2.00 (0.045) | -0.83 (0.405) | -0.03 (0.976) |

All of the displayed *P* values were the original ones. lmt_scr_perc_correct, Percentage correct of all 32 presented trials; lmt_scr_perc_wrong, Percentage wrong (including timed out) out of 32 trials; lmt_scr_num_correct, Number correct; lmt_scr_num_wrong, Number wrong, not including timed out; pea_ravlt_sd_trial_i_tc, RAVLT Short Delay Trial I Total Correct; pea_ravlt_sd_trial_ii_tc, RAVLT Short Delay Trial II Total Correct; pea_ravlt_sd_trial_iii_tc, RAVLT Short Delay Trial III Total Correct; pea_ravlt_sd_trial_iv_tc, RAVLT Short Delay Trial IV Total Correct; pea_ravlt_sd_trial_v_tc, RAVLT Short Delay Trial V Total Correct; pea_ravlt_sd_listb_tc, RAVLT Short Delay List B Total Correct; pea_ravlt_sd_trial_vi_tc, RAVLT Short Delay Trial VI Total Correct; pea_ravlt_ld_trial_vii_tc, RAVLT Long Delay VII Total Correct; pea_wiscv_trs, WISC-V Matrix Reasoning Total Raw Score; pea_wiscv_tss, WISC-V Matrix Reasoning Total Scaled Score.

**Table S4.** Group difference for gestational age and cognitive measures at 11-12 years

| Cognition | Main Effect F (*P*) values | Simple Effect t (*P*) values | | | |
| --- | --- | --- | --- | --- | --- |
|  |  | ≤33weeks | 34~35weeks | 36weeks | 37~39weeks |
| nihtbx_totalcomp_fc | n.a. | n.a. | n.a. | n.a. | n.a. |
| nihtbx_cryst_fc | **9.11 (2.5×10^-7^)** | -4.82 (1.5×10^-7^) | -3.29 (0.001) | -2.29 (0.02) | -1.39 (0.16) |
| N | **-** | 254 | 328 | 327 | 315 |
| nihtbx_fluidcomp_fc | n.a. | n.a. | n.a. | n.a. | n.a. |
| nihtbx_reading_fc | **8.63 (6.1×10^-7^)** | -4.44 (9×10^-6^) | -3.48 (5×10^-4^) | -2.2 (0.03) | -1.67 (0.09) |
| N | **-** | 254 | 325 | 319 | 306 |
| nihtbx_picvocab_fc | **4.92 (6×10^-4^)** | -3.92 (9×10^-5^) | -1.85 (0.06) | -1.55 (0.12) | -0.79 (0.43) |
| N | **-** | 256 | 326 | 320 | 307 |
| nihtbx_list_fc | n.a. | n.a. | n.a. | n.a. | n.a. |
| nihtbx_picturce_fc | **4.12 (0.002)** | -3.19 (0.001) | -2.22 (0.03) | -1.39 (0.16) | 0.58 (0.56) |
| N | **-** | 255 | 327 | 326 | 315 |
| nihtbx_flanker_fc | **5.06 (5×10^-4^)** | -3.06 (0.002) | -1.43 (0.15) | -3.24 (0.001) | -0.97 (0.33) |
| N | **-** | 255 | 329 | 328 | 316 |
| nihtbx_cardsort_fc | n.a. | n.a. | n.a. | n.a. | n.a. |
| nihtbx_pattern_fc | 3.1 (0.015) | -2.58 (0.01) | 0.01 (0.99) | -2.51 (0.01) | -0.03 (0.97) |
| N | - | 256 | 329 | 327 | 315 |

Gestational age of more than 40 weeks was taken as the reference group in the simple effect test. All of the displayed *P* values were the original ones. Bold values indicated significance with Bonferroni correction (*P*<0.05). Note: No multiple comparison correction was applied to the simple effect test because it is a post-hoc analysis following main effect detection. Abbreviations: nihtbx_totalcomp_fc: Cognition Total Composite Score Fully-Corrected T-score; nihtbx_cryst_fc: Crystallized Composite Fully-Corrected T-score; nihtbx_fluidcomp_fc: Cognition Fluid Composite Fully-Corrected T-score; nihtbx_reading_fc: NIH Toolbox Oral Reading Recognition Test Age 3+ v2.0 Fully-Corrected T-score; nihtbx_picvocab_fc: NIH Toolbox Picture Vocabulary Test Age 3+ v2.0 Fully-Corrected T-score; nihtbx_list_fc: NIH Toolbox List Sorting Working Memory Test Age 7+ v2.0 Fully-Corrected T-score; nihtbx_picture_fc: NIH Toolbox Picture Sequence Memory Test Age 8+ Form A v2.0 Fully-Corrected T-score; nihtbx_flanker_fc: NIH Toolbox Flanker Inhibitory Control and Attention Test Ages 8-11 v2.0 Fully-Corrected T-score; nihtbx_cardsort_fc: NIH Toolbox Dimensional Change Card Sort Test Ages 8-11 v2.0 Fully-Corrected T-score; nihtbx_pattern_fc: NIH Toolbox Pattern Comparison Processing Speed Test Age 7+ v2.0 Fully-Corrected T-score. n.a., not available because of lots of missing data. N, number of subjects.

**Table S5.** Group difference for the association between gestational age and brain volume in adolescents at 9-10 years

| Volumetric regions | Main Effect  F (*P*) values | Simple Effect  t (*P*) values | | | |
| --- | --- | --- | --- | --- | --- |
|  |  | ≤33 week | 34~35 week | 36 week | 37~39 week |
| bankssts_lh | **6.79 (1.9×10^-5^)** | **-4.14 (3.5×10^-5^)** | -3.00 (0.002) | -1.27 (0.21) | -1.63 (0.10) |
| fusiform_lh | **7.64 (3.8×10^-6^)** | **-5.03 (5.0×10^-7^)** | -2.20 (0.03) | -1.40 (0.16) | -1.78 (0.08) |
| inferior parietal_lh | **14.2 (1.6×10^-11^)** | **-5.87 (4.4×10^-9^)** | **-4.54 (5.73×10^-6^)** | -2.56 (0.01) | -2.54 (0.01) |
| middle temporal_lh | **15.3 (1.7×10^-12^)** | **-7.1 (1.3×10^-12^)** | **-3.47 (5.0×10^-4^)** | -1.65 (0.10) | -2.25 (0.02) |
| pars orbitalis_lh | **5.08 (4.0×10^-4^)** | **-3.57 (4.0×10^-4^)** | -1.88 (0.06) | -2.43 (0.02) | -0.16 (0.87) |
| pars triangularis_lh | **7.59 (4.2×10^-6^)** | **-4.33 (1.5×10^‑5^)** | -2.83 (0.005) | -2.35 (0.02) | 0.44 (0.66) |
| postcentral_lh | **13.4 (7.4×10^-11^)** | **-5.6 (2.1×10^-8^)** | -2.74 (0.006) | -1.07 (0.28) | **-4.65 (3.36×10^-6^)** |
| rostral anterior cingulate_lh | **8.81 (4.2×10^-7^)** | **-4.74 (2.2×10^-6^)** | -2.98 (0.003) | -2.23 (0.03) | -2.53 (0.01) |
| superior parietal_lh | **5.59 (2.0×10^-4^)** | **-4.02 (5.8×10^-5^)** | -2.83 (0.005) | -0.73 (0.46) | -0.59 (0.56) |
| insula_lh | **6.29 (4.8×10^-5^)** | **-3.63 (3.0×10^-4^)** | -1.40 (0.16) | -1.89 (0.06) | -3.34 (8.0×10^-4^) |
| bankssts_rh | **9.32 (1.6×10^-7^)** | **-4.22 (2.4×10^-5^)** | **-3.46 (5.0×10^-4^)** | -2.58 (0.01) | -3.08 (0.002) |
| caudal middle frontal_rh | **6.51 (3.0×10^-5^)** | **-4.23 (2.3×10^-5^)** | -2.31 (0.02) | 0.65 (0.52) | -2.08 (0.04) |
| fusiform_rh | **7.28 (7.5×10^-6^)** | **-4.41 (1.1×10^-5^)** | -3.05 (0.002) | -1.14 (0.26) | -1.52 (0.13) |
| inferior parietal_rh | **11.3 (4.1×10^-9^)** | **-5.25 (1.6×10^-7^)** | **-3.57 (4.0×10^-4^)** | -1.58 (0.11) | -3.29 (0.001) |
| isthmus cingulate_rh | **6.25 (5.1×10^-5^)** | **-4.24 (2.2×10^-5^)** | -2.37 (0.02) | -1.79 (0.07) | -1.69 (0.09) |
| middle temporal_rh | **24.1 (6.8×10^-20^)** | **-8.56 (1.3×10^-17^)** | **-4.67 (3.1×10^-6^)** | -2.58 (0.01) | -3.34 (8.0×10^-4^) |
| pars orbitalis_rh | **6.35 (4.0×10^-5^)** | **-3.84 (1.0×10^-4^)** | -3.02 (0.002) | -2.40 (0.02) | -0.52 (0.60) |
| pars triangularis_rh | **8.23 (1.3×10^-6^)** | **-4.51 (6.0×10^-6^)** | -1.66 (0.10) | **-3.55 (4.0×10^-4^)** | -0.55 (0.58) |
| postcentral_rh | **7.96 (2.1×10^-6^)** | **-5.21 (1.9×10^-7^)** | -1.61 (0.11) | -1.64 (0.10) | -1.96 (0.05) |
| posterior cingulate_rh | **5.34 (3.0×10^-4^)** | **-3.78 (2.0×10^-4^)** | -2.58 (0.01) | -0.52 (0.60) | -1.49 (0.14) |
| precuneus_rh | **5.56 (2.0×10^-4^)** | **-4.40 (1.1×10^-5^)** | -1.53 (0.13) | -0.69 (0.49) | -1.35 (0.18) |
| superior frontal_rh | **5.40 (2.0×10^-4^)** | **-4.12 (3.8×10^-5^)** | -0.79 (0.43) | -1.36 (0.17) | -2.24 (0.03) |
| supramarginal_rh | **8.67 (5.6×10^-7^)** | **-4.74 (2.2×10^-6^)** | **-3.49 (5.0×10^-4^)** | -2.28 (0.02) | -1.20 (0.23) |
| transverse temporal_rh | **5.20 (3.0×10^-4^)** | **-4.09 (4.4×10^-5^)** | -2.25 (0.02) | -0.81 (0.42) | -1.13 (0.26) |
| insula_rh | **7.21 (8.5×10^-6^)** | **-3.98 (7.0×10^-5^)** | -1.18 (0.24) | -3.02 (0.003) | -2.85 (0.004) |
| thalamus proper_rh | **12.7 (2.4×10^-10^)** | **-6.82 (9.6×10^-12^)** | -2.53 (0.01) | -0.36 (0.72) | -1.44 (0.15) |
| pallidum_rh | **5.05 (4.6×10^-4^)** | **-3.51 (4.5×10^-4^)** | **-3.11 (0.002)** | -0.77 (0.44) | -0.71 (0.48) |
| hippocampus_rh | **6.75 (2.0×10^-5^)** | **-5.52 (2.6×10^-7^)** | -0.53 (0.60) | -0.22 (0.83) | -0.99 (0.32) |
| total left hemisphere | 9.21 (2.0×10^-7^) | -5.19 (2.1×10^-7^) | -2.41 (0.02) | -1.39 (0.17) | -2.87 (0.004) |
| total right hemisphere | 9.34 (1.6×10^-7^) | -5.36 (8.4×10^-8^) | -2.36 (0.02) | -1.65 (0.10) | -2.56 (0.01) |
| total brain volume | 9.31 (1.7×10^-7^) | -5.29 (1.2×10^-7^) | -2.39 (0.02) | -1.52 (0.13) | -2.73 (0.006) |

Gestational age of more than 40 weeks was taken as reference group in the simple effect test. All of the displayed *P* values are the original ones. Bold values indicated significance with Bonferroni correction (*P*<0.05). Note: No multiple comparison correction was applied to the simple effect test because it is a post-hoc analysis following main effect detection. Abbreviations: banksstsrh: banks of superior temporal sulcus; lh, left hemisphere; rh, right hemisphere.

**Table S6.** Mediation result for mediation by each volumetric region of the association between gestational age and cognitive measures in adolescents at 9-10 years

| **total composite cognitive score** | | | | | |
| --- | --- | --- | --- | --- | --- |
| Volumetric regions | Path A  Beta (*P*) values | Path B  Beta (*P*) values | Path C’  Beta (*P*) values | Path C  Beta (*P*) values | Path AB  Beta (*P*) values |
| bankssts_lh | 0.039(0.0002) | 0.013(0.179) | 0.044(0.0002) | 0.045(0.0002) | 0.001(0.137) |
| fusiform_lh | 0.049(0.00023) | 0.036(0.0002) | 0.043(0.00019) | 0.045(0.00019) | 0.002(0.0001) |
| inferior parietal_lh | 0.071(0.00023) | 0.032(0.0013) | 0.043(0.00016) | 0.045(0.00016) | 0.002(0.0009) |
| middle temporal_lh | 0.071(0.00021) | 0.033(0.001) | 0.043(0.00018) | 0.045(0.00018) | 0.002(0.0008) |
| pars orbitalis_lh | 0.046(0.00017) | 0.014(0.171) | 0.044(0.00023) | 0.045(0.00023) | 0.001(0.139) |
| pars triangularis_lh | 0.04(0.00021) | 0.006(0.566) | 0.045(0.00019) | 0.045(0.00019) | 0.0002(0.496) |
| postcentral_lh | 0.056(0.0002) | 0.014(0.171) | 0.044(0.00023) | 0.045(0.00023) | 0.001(0.142) |
| rostral anterior cingulate_lh | 0.055(0.0002) | 0.039(0.00018) | 0.043(0.00017) | 0.045(0.00017) | 0.002(0.0001) |
| superior parietal_lh | 0.044(0.00023) | 0.004(0.717) | 0.045(0.00023) | 0.045(0.00023) | 0.00016(0.688) |
| insula_lh | 0.041(0.00024) | 0.044(0.00025) | 0.043(0.00019) | 0.045(0.0002) | 0.002(0.0001) |
| bankssts_rh | 0.049(0.00024) | 0.019(0.054) | 0.044(0.00018) | 0.045(0.00018) | 0.001(0.037) |
| caudal middle frontal_rh | 0.042(0.0002) | 0.026(0.0099) | 0.044(0.00023) | 0.045(0.00022) | 0.001(0.006) |
| fusiform_rh | 0.05(0.0002) | 0.029(0.0027) | 0.044(0.0002) | 0.045(0.0002) | 0.001(0.002) |
| inferior parietal_rh | 0.062(0.00022) | 0.025(0.013) | 0.043(0.00023) | 0.045(0.00023) | 0.002(0.0094) |
| isthmus cingulate_rh | 0.043(0.00027) | 0.032(0.0008) | 0.044(0.00019) | 0.045(0.00018) | 0.001(0.0005) |
| middle temporal_rh | 0.087(0.00024) | 0.029(0.004) | 0.042(0.00021) | 0.045(0.00021) | 0.003(0.003) |
| pars orbitalis_rh | 0.05(0.00019) | 0.011(0.260) | 0.044(0.00023) | 0.045(0.00023) | 0.001(0.232) |
| pars triangularis_rh | 0.039(0.00023) | -0.004(0.668) | 0.045(0.00018) | 0.045(0.00018) | -0.00015(0.711) |
| postcentral_rh | 0.048(0.00022) | 0.013(0.178) | 0.044(0.00019) | 0.045(0.00019) | 0.001(0.148) |
| posterior cingulate_rh | 0.033(0.00098) | 0.008(0.400) | 0.045(0.00021) | 0.045(0.00021) | 0.00027(0.324) |
| precuneus_rh | 0.037(0.00017) | 0.028(0.003) | 0.044(0.00021) | 0.045(0.00021) | 0.001(0.0017) |
| superior frontal_rh | 0.039(0.00017) | 0.028(0.003) | 0.044(0.00023) | 0.045(0.00023) | 0.001(0.0017) |
| supramarginal_rh | 0.056(0.00024) | 0.033(0.001) | 0.043(0.00022) | 0.045(0.00022) | 0.002(0.0009) |
| transverse temporal_rh | 0.048(0.0002) | 0.013(0.209) | 0.044(0.00021) | 0.045(0.0002) | 0.001(0.167) |
| insula_rh | 0.047(0.00022) | 0.05(0.0002) | 0.043(0.00023) | 0.045(0.00023) | 0.002(0.0001) |
| thalamus proper_rh | 0.062(0.00017) | 0.05(0.0002) | 0.042(0.00024) | 0.045(0.00024) | 0.003(0.00012) |
| pallidum_rh | 0.039(0.00017) | 0.041(0.0002) | 0.043(0.00022) | 0.045(0.00021) | 0.002(0.0001) |
| hippocampus_rh | 0.038(0.00018) | 0.036(0.0002) | 0.044(0.0002) | 0.045(0.0002) | 0.001(0.00021) |
| total left hemisphere | 0.055(0.00019) | 0.036(0.0002) | 0.043(0.00021) | 0.045(0.00021) | 0.002(0.00012) |
| total right hemisphere | 0.055(0.00021) | 0.035(0.0005) | 0.043(0.00022) | 0.045(0.00021) | 0.002(0.00036) |
| total brain volume | 0.056(0.00021) | 0.036(0.0004) | 0.043(0.00019) | 0.045(0.00019) | 0.002(0.00024) |
| **crystallized composite score** | | | | | |
| Brain region | Path A  Beta (*P*) values | Path B  Beta (*P*) values | Path C’  Beta (*P*) values | Path C  Beta (*P*) values | Path AB  Beta (*P*) values |
| bankssts_lh | 0.039(0.0002) | 0.013(0.179) | 0.044(0.0002) | 0.045(0.0002) | 0.001(0.1374) |
| fusiform_lh | 0.049(0.0002) | 0.036(0.0002) | 0.043(0.00019) | 0.045(0.00019) | 0.002(0.0001) |

**Table S6.** Mediation result for mediation by each volumetric region for the association between gestational age and cognitive measures in adolescents at 9-10 years (continued)

| **crystallized composite score** | | | | | |
| --- | --- | --- | --- | --- | --- |
| Volumetric regions | Path A  Beta (*P*) values | Path B  Beta (*P*) values | Path C’  Beta (*P*) values | Path C  Beta (*P*) values | Path AB  Beta (*P*) values |
| inferior parietal_lh | 0.071(0.0002) | 0.032(0.001) | 0.043(0.00016) | 0.045(0.00016) | 0.002(0.0009) |
| middle temporal_lh | 0.071(0.0002) | 0.033(0.001) | 0.043(0.00018) | 0.045(0.00018) | 0.002(0.0008) |
| pars orbitalis_lh | 0.046(0.00017) | 0.014(0.171) | 0.044(0.0002) | 0.045(0.00023) | 0.001(0.1392) |
| pars triangularis_lh | 0.04(0.00021) | 0.006(0.566) | 0.045(0.00019) | 0.045(0.00019) | 0.0006(0.497) |
| Postcentral_lh | 0.056(0.0002) | 0.014(0.171) | 0.044(0.0002) | 0.045(0.00023) | 0.001(0.142) |
| rostral anterior cingulate_lh | 0.055(0.0002) | 0.039(0.0002) | 0.043(0.00018) | 0.045(0.00017) | 0.002(0.0001) |
| superior parietal_lh | 0.044(0.0002) | 0.004(0.717) | 0.045(0.0002) | 0.045(0.00023) | 0.0008(0.688) |
| insula_lh | 0.041(0.0002) | 0.044(0.00025) | 0.043(0.0002) | 0.045(0.0002) | 0.002(0.00012) |
| bankssts_rh | 0.049(0.0002) | 0.019(0.0536) | 0.044(0.0002) | 0.045(0.00018) | 0.001(0.0372) |
| caudal middle frontal_rh | 0.042(0.0002) | 0.026(0.0099) | 0.044(0.0002) | 0.045(0.00022) | 0.001(0.0058) |
| fusiform_rh | 0.05(0.0002) | 0.029(0.0028) | 0.044(0.0002) | 0.045(0.0002) | 0.001(0.0016) |
| inferior parietal_rh | 0.062(0.0002) | 0.025(0.013) | 0.043(0.00023) | 0.045(0.00023) | 0.002(0.0095) |
| isthmus cingulate_rh | 0.043(0.00027) | 0.032(0.001) | 0.044(0.00019) | 0.045(0.00018) | 0.001(0.0005) |
| middle temporal_rh | 0.087(0.00024) | 0.029(0.004) | 0.042(0.0002) | 0.045(0.00021) | 0.003(0.0031) |
| pars orbitalis_rh | 0.05(0.00019) | 0.011(0.260) | 0.044(0.00023) | 0.045(0.00023) | 0.001(0.232) |
| pars triangularis_rh | 0.039(0.00023) | -0.004(0.668) | 0.045(0.00018) | 0.045(0.00018) | -0.0001(0.711) |
| postcentral_rh | 0.048(0.00022) | 0.013(0.178) | 0.044(0.00019) | 0.045(0.00019) | 0.0006(0.148) |
| posterior cingulate_rh | 0.033(0.00098) | 0.008(0.400) | 0.045(0.00021) | 0.045(0.00021) | 0.0003(0.324) |
| precuneus_rh | 0.037(0.00017) | 0.028(0.003) | 0.044(0.00021) | 0.045(0.0002) | 0.001(0.0017) |
| superior frontal_rh | 0.039(0.00017) | 0.028(0.003) | 0.044(0.00023) | 0.045(0.00023) | 0.001(0.0017) |
| supramarginal_rh | 0.056(0.00024) | 0.033(0.001) | 0.043(0.00022) | 0.045(0.00022) | 0.0018(0.0009) |
| transverse temporal_rh | 0.048(0.0002) | 0.013(0.209) | 0.044(0.00021) | 0.045(0.0002) | 0.0006(0.167) |
| insula_rh | 0.047(0.0002) | 0.05(0.0002) | 0.043(0.00023) | 0.045(0.00023) | 0.002(9.9×10^-5^) |
| thalamus proper_rh | 0.062(0.00017) | 0.05(0.0002) | 0.042(0.00024) | 0.045(0.00024) | 0.003(0.00012) |
| pallidum_rh | 0.039(0.00017) | 0.041(0.0002) | 0.043(0.00022) | 0.045(0.00021) | 0.002(0.0001) |
| hippocampus_rh | 0.038(0.00018) | 0.036(0.0002) | 0.044(0.0002) | 0.045(0.00020) | 0.0014(0.0002) |
| total left hemisphere | 0.055(0.00019) | 0.036(0.0002) | 0.043(0.00021) | 0.045(0.00021) | 0.002(0.0001) |
| total right hemisphere | 0.055(0.0002) | 0.035(0.0005) | 0.043(0.00022) | 0.045(0.00021) | 0.002(0.0004) |
| total brain volume | 0.056(0.0002) | 0.036(0.00037) | 0.043(0.00019) | 0.045(0.00019) | 0.002(0.0002) |
| **fluid composite score** | | | | | |
| Volumetric regions | Path A  Beta (*P*) values | Path B  Beta (*P*) values | Path C’  Beta (*P*) values | Path C  Beta (*P*) values | Path AB  Beta (*P*) values |
| bankssts_lh | 0.039(0.0002) | 0.013(0.179) | 0.044(0.0002) | 0.045(0.0002) | 0.0005(0.137) |
| fusiform_lh | 0.049(0.00023) | 0.036(0.0002) | 0.043(0.00019) | 0.045(0.00019) | 0.0017(0.0001) |
| inferior parietal_lh | 0.071(0.00023) | 0.032(0.001) | 0.043(0.00016) | 0.045(0.00016) | 0.0023(0.0009) |
| middle temporal_lh | 0.071(0.00021) | 0.033(0.001) | 0.043(0.00018) | 0.045(0.00018) | 0.0023(0.0008) |
| pars orbitalis_lh | 0.046(0.00017) | 0.014(0.171) | 0.044(0.00023) | 0.045(0.00023) | 0.0006(0.139) |
| pars triangularis_lh | 0.04(0.00021) | 0.006(0.566) | 0.045(0.00019) | 0.045(0.00019) | 0.0002(0.496) |

**Table S6.** Mediation result for mediation by each volumetric region for the association between gestational age and cognitive performance in adolescents at 9-10 years (continued)

| **fluid composite score** | | | | | |
| --- | --- | --- | --- | --- | --- |
| Volumetric regions | Path A  Beta (*P*) values | Path B  Beta (*P*) values | Path C’  Beta (*P*) values | Path C  Beta (*P*) values | Path AB  Beta (*P*) values |
| postcentral_lh | 0.056(0.0002) | 0.014(0.171) | 0.044(0.0002) | 0.045(0.00023) | 0.0007(0.142) |
| rostral anterior cingulate_lh | 0.055(0.0002) | 0.039(0.0002) | 0.043(0.00017) | 0.045(0.00017) | 0.0022(0.0001) |
| superior parietal_lh | 0.044(0.00023) | 0.004(0.717) | 0.045(0.00023) | 0.045(0.00023) | 0.0002(0.688) |
| insula_lh | 0.041(0.00024) | 0.044(0.0003) | 0.043(0.00019) | 0.045(0.0002) | 0.0018(0.0001) |
| bankssts_rh | 0.049(0.00024) | 0.019(0.0536) | 0.044(0.00018) | 0.045(0.00018) | 0.0009(0.0372) |
| caudal middle frontal_rh | 0.042(0.0002) | 0.026(0.010) | 0.044(0.00023) | 0.045(0.0002) | 0.0011(0.006) |
| fusiform_rh | 0.05(0.0002) | 0.029(0.003) | 0.044(0.0002) | 0.045(0.0002) | 0.0015(0.0016) |
| inferior parietal_rh | 0.062(0.0002) | 0.025(0.0129) | 0.043(0.00023) | 0.045(0.0002) | 0.0016(0.009) |
| isthmus cingulate_rh | 0.043(0.00027) | 0.032(0.0008) | 0.044(0.00019) | 0.045(0.00018) | 0.0014(0.0005) |
| middle temporal_rh | 0.087(0.00024) | 0.029(0.004) | 0.042(0.00021) | 0.045(0.0002) | 0.0025(0.003) |
| pars orbitalis_rh | 0.05(0.00019) | 0.011(0.260) | 0.044(0.00023) | 0.045(0.0002) | 0.0005(0.232) |
| pars triangularis_rh | 0.039(0.00023) | -0.004(0.668) | 0.045(0.00018) | 0.045(0.00018) | -0.0001(0.711) |
| postcentral_rh | 0.048(0.00022) | 0.013(0.178) | 0.044(0.00019) | 0.045(0.00019) | 0.0006(0.148) |
| posterior cingulate_rh | 0.033(0.0010) | 0.008(0.400) | 0.045(0.0002) | 0.045(0.00021) | 0.0003(0.324) |
| precuneus_rh | 0.037(0.00017) | 0.028(0.003) | 0.044(0.0002) | 0.045(0.0002) | 0.001(0.0018) |
| superior frontal_rh | 0.039(0.00017) | 0.028(0.003) | 0.044(0.00023) | 0.045(0.0002) | 0.001(0.0017) |
| supramarginal_rh | 0.056(0.00024) | 0.033(0.001) | 0.043(0.0002) | 0.045(0.0002) | 0.0018(0.0009) |
| transverse temporal_rh | 0.048(0.0002) | 0.013(0.209) | 0.044(0.00021) | 0.045(0.0002) | 0.0006(0.167) |
| insula_rh | 0.047(0.0002) | 0.05(0.0002) | 0.043(0.00023) | 0.045(0.0002) | 0.002(9.9×10^-5^) |
| thalamus proper_rh | 0.062(0.00017) | 0.05(0.0002) | 0.042(0.00024) | 0.045(0.00023) | 0.003(0.0001) |
| pallidum_rh | 0.039(0.00017) | 0.041(0.0002) | 0.043(0.00022) | 0.045(0.00021) | 0.0016(0.0001) |
| hippocampus_rh | 0.038(0.00018) | 0.036(0.0002) | 0.044(0.0002) | 0.045(0.0002) | 0.0014(0.0002) |
| total left hemisphere | 0.055(0.00019) | 0.036(0.0002) | 0.043(0.0002) | 0.045(0.0002) | 0.002(0.0001) |
| total right hemisphere | 0.055(0.0002) | 0.035(0.0005) | 0.043(0.0002) | 0.045(0.0002) | 0.002(0.0004) |
| total brain volume | 0.056(0.0002) | 0.036(0.0004) | 0.043(0.00019) | 0.045(0.00019) | 0.002(0.0002) |

Abbreviations: banksstsrh: banks of superior temporal sulcus; lh, left hemisphere; rh, right hemisphere. All of the displayed *P* values were the original ones.

**Table S7.** Longitudinal group-by-time interaction for brain volume in adolescents from 9-10 years to 11-12 years

| Volumetric regions | Gestational Age | | | | | | | | | |  | |
| --- | --- | --- | --- | --- | --- | --- | --- | --- | --- | --- | --- | --- |
|  | ≤33 week | 34~35 week | 36 week | 37~39 week | 40 week | ≤33 week | 34~35 week | 36 week | 37~39 week | 40 week | F values | P values |
|  | Baseline (9-10 years) | | | | | 2-years follow-up (11-12 years) | | | | |  |  |
| bankssts_lh | 3.28 (0.63) | 3.32 (0.59) | 3.33 (0.58) | 3.34 (0.56) | 3.37 (0.59) | 3.24 (0.63) | 3.28 (0.59) | 3.278 (0.58) | 3.26 (0.54) | 3.30 (0.58) | 0.06 | 0.99 |
| fusiform_lh | 12.1 (1.82) | 12.3 (1.63) | 12.2 (1.67) | 12.4 (1.71) | 12.4 (1.70) | 11.8 (1.82) | 12.1 (1.61) | 11.9 (1.64) | 12.2 (1.68) | 12.2 (1.71) | 0.49 | 0.75 |
| inferior parietal_lh | 16.3 (2.43) | 16.4 (2.25) | 16.5 (2.34) | 16.6 (2.45) | 16.8 (2.30) | 15.8 (2.39) | 16.0 (2.27) | 16.0 (2.31) | 16.1 (2.41) | 16.3 (2.29) | 0.14 | 0.97 |
| middle temporal_lh | 13.3 (1.91) | 13.6 (1.78) | 13.7 (1.86) | 13.7 (1.79) | 13.7 (1.80) | 13.1 (1.94) | 13.4 (1.81) | 13.5 (1.81) | 13.6 (1.78) | 13.6 (1.82) | 0.11 | 0.98 |
| pars orbitalis_lh | 2.81 (0.38) | 2.81 (0.39) | 2.80 (0.37) | 2.87 (0.36) | 2.83 (0.39) | 2.75 (0.37) | 2.76 (0.39) | 2.75 (0.36) | 2.81 (0.35) | 2.78 (0.38) | 0.67 | 0.61 |
| pars triangularis_lh | 4.67 (0.80) | 4.73 (0.77) | 4.70 (0.71) | 4.90 (0.76) | 4.79 (0.77) | 4.55 (0.77) | 4.63 (0.75) | 4.58 (0.73) | 4.78 (0.75) | 4.69 (0.76) | 0.81 | 0.52 |
| postcentral_lh | 12.1 (1.79) | 12.1 (1.71) | 12.3 (1.83) | 12.3 (1.72) | 12.3 (1.80) | 11.8 (1.85) | 11.8 (1.62) | 11.9 (1.75) | 11.9 (1.69) | 12.0 (1.77) | 0.64 | 0.63 |
| rostral anterior cingulate_lh | 3.32 (0.62) | 3.31 (0.62) | 3.31 (0.60) | 3.30 (0.53) | 3.34 (0.60) | 3.28 (0.61) | 3.31 (0.61) | 3.25 (0.59) | 3.31 (0.55) | 3.30 (0.59) | 0.57 | 0.68 |
| superior parietal_lh | 16.9 (2.08) | 16.8 (2.21) | 17.1 (2.13) | 17.1 (2.12) | 17.1 (2.28) | 16.3 (2.73) | 16.3 (2.18) | 16.5 (2.08) | 16.6 (2.08) | 16.6 (2.22) | 0.30 | 0.88 |
| insula_lh | 7.51 (0.88) | 7.59 (0.86) | 7.55 0.83) | 7.59 (0.89) | 7.63 0.87) | 7.38 (0.86) | 7.60 (0.91) | 7.48 (0.79) | 7.55 (0.90) | 7.57 (0.87) | 0.43 | 0.79 |
| bankssts_rh | 3.09 (0.55) | 3.10 (0.49) | 3.12 (0.51) | 3.12 (0.51) | 3.18 (0.52) | 3.30 (0.53) | 3.07 (0.50) | 3.05 (0.50) | 3.07 (0.51) | 3.13 (0.51) | 0.70 | 0.59 |
| caudal middle frontal_rh | 7.79 (1.55) | 7.80 (1.61) | 8.13 (1.63) | 8.15 (1.63) | 8.08 (1.57) | 7.79 (1.49) | 7.75 (1.60) | 8.04 (1.59) | 8.03 (1.64) | 7.99 (1.54) | 0.25 | 0.91 |
| fusiform_rh | 11.7 (1.73) | 11.9 (1.58) | 11.9 (1.71) | 12.0 (1.61) | 11.9 (1.63) | 11.5 (1.71) | 11.7 (1.60) | 11.7 (1.60) | 11.8 (1.58) | 11.7 (1.63) | 0.62 | 0.65 |
| inferior parietal_rh | 19.8 (3.00) | 20.0 (2.55) | 20.1 (2.82) | 20.1 (2.80) | 20.3 (2.65) | 19.3 (2.94) | 19.5 (2.60) | 19.5 (2.78) | 19.6 (2.75) | 19.8 (2.66) | 0.52 | 0.72 |
| isthmus cingulate_rh | 3.11 (0.59) | 3.11 (0.61) | 3.08 (0.62) | 3.14 (0.59) | 3.16 (0.59) | 3.37 (0.59) | 3.05 (0.59) | 2.99 (0.59) | 3.06 (0.58) | 3.08 (0.58) | 0.43 | 0.79 |
| middle temporal_rh | 14.6 (1.97) | 15.0 (1.89) | 14.9 (1.94) | 15.1 (1.90) | 15.1 (1.94) | 14.5 (2.90) | 14.8 (1.85) | 14.7 (1.90) | 15.0 (1.90) | 15.0 (1.93) | 0.30 | 0.88 |
| pars orbitalis_rh | 3.38 (0.47) | 3.40 (0.45) | 3.38 (0.45) | 3.46 (0.48) | 3.41 (0.46) | 3.33 (0.47) | 3.31 (0.44) | 3.37 (0.45) | 3.39 (0.45) | 3.34 (0.46) | 0.37 | 0.83 |
| pars triangularis_rh | 5.58 (0.10) | 5.61 (0.95) | 5.50 (0.99) | 5.71 (0.88) | 5.63 (0.92) | 5.47 (1.60) | 5.49 (0.92) | 5.38 (0.97) | 5.61 (0.89) | 5.52 (0.91) | 0.67 | 0.61 |
| postcentral_rh | 11.2 (1.47) | 11.4 (1.67) | 11.4 (1.65) | 11.5 (1.57) | 11.5 (1.70) | 10.9 (1.56) | 11.1 (1.59) | 11.1 (1.61) | 11.2 (1.53) | 11.1 (1.66) | 1.10 | 0.36 |
| posterior cingulate_rh | 4.17 (0.72) | 4.24 (0.71) | 4.25 (0.75) | 4.25 (0.80) | 4.25 (0.72) | 4.64 (0.69) | 4.15 (0.71) | 4.09 (0.70) | 4.15 (0.75) | 4.14 (0.70) | 0.07 | 0.99 |
| precuneus_rh | 13.1 (1.75) | 13.1 (1.69) | 13.1 (1.69) | 13.3 (1.80) | 13.2 (1.75) | 12.8 (1.76) | 12.7 (1.67) | 12.8 (1.67) | 12.9 (1.76) | 12.8 (1.73) | 0.21 | 0.93 |
| superior frontal_rh | 28.0 (3.37) | 28.3 (3.30) | 28.2 (3.31) | 28.3 (3.08) | 28.4 (3.26) | 27.7 (3.31) | 27.9 (3.27) | 27.8 (3.36) | 28.0 (3.08) | 28.0 (0.33) | 0.24 | 0.92 |
| supramarginal_rh | 13.2 (1.78) | 13.3 (1.94) | 13.3 (1.92) | 13.5 (1.82) | 13.4 (2.04) | 12.9 (1.85) | 12.9 (1.90) | 13.0 (1.89) | 13.2 (1.84) | 13.1 (2.00) | 0.77 | 0.55 |
| transverse temporal_rh | 1.14 (0.23) | 1.15 (0.23) | 1.15 (0.21) | 1.17 (0.21) | 1.16 (0.22) | 1.12 (0.22) | 1.12 (0.22) | 1.13 (0.21 | 1.14 (0.21 | 1.14 (0.21) | 0.30 | 0.88 |
| insula_rh | 7.47 (0.91) | 7.58 (0.86) | 7.57 (0.88) | 7.66 (0.96) | 7.64 (0.89) | 7.41 (0.94) | 7.58 (0.87) | 7.56 (0.91) | 7.61 (0.99) | 7.61 (0.90) | 0.93 | 0.44 |
| thalamus proper_rh | 7.14 (0.74) | 7.23 (0.73) | 7.29 (0.69) | 7.30 (0.74) | 7.28 (0.70) | 7.27 (0.76) | 7.35 (0.74) | 7.41 (0.69) | 7.44 (0.75) | 7.40 (0.72) | 0.06 | 0.99 |
| pallidum_rh | 1.64 (0.20) | 1.63 (0.19) | 1.64 (0.19) | 1.66 (0.21) | 1.66 (0.20) | 1.64 (0.20) | 1.63 (0.18) | 1.64 (0.18) | 1.67 (0.21) | 1.66 (0.20) | 0.49 | 0.75 |
| hippocampus_rh | 4.05 (0.50) | 4.16 (0.46) | 4.13 (0.42) | 4.13 (0.45) | 4.09 (0.43) | 4.15 (0.44) | 4.21 (0.45) | 4.18 (0.43) | 4.20 (0.47) | 4.16 (0.43) | 0.14 | 0.97 |
| total left hemisphere | 296.7 (29.2) | 297.5(27.2) | 298.3 (28.1) | 298.9 (27.2) | 298.7 (28.5) | 290.6 (29.7) | 292.5 (27.2) | 292.1 (27.6) | 293.5 (27.8) | 293.2 (28.8) | 0.06 | 0.99 |
| total right hemisphere | 297 (28.6) | 298.2(27.2) | 298.6 (28.3) | 299.9 (27.3) | 299.6 (28.6) | 292.0 (29.4) | 292.7 (27.5) | 292.4 (27.9) | 294.3 (27.9) | 293.9 (29.0) | 0.02 | 0.99 |
| total brain volume | 594 (57.7) | 595.8 (54.2) | 596.9 (56.4) | 598.8 (54.4) | 598.4 (57.0) | 581.7 (59.4) | 585.3 (54.6) | 584.5 (55.4) | 587.8 (55.6) | 587.1 (57.7) | 0.04 | 0.99 |

The values represented mean (std) cm^3^. Abbreviations: banksstsrh: banks of superior temporal sulcus; lh, left hemisphere; rh, right hemisphere.

**Table S8.** Group difference for the association between gestational age and brain volume when excluding adolescents with extreme birth weight at 9-10 years

| Volumetric regions | Main Effect  F (*P*) values | Simple Effect  T (*P*) values | | | |
| --- | --- | --- | --- | --- | --- |
|  |  | ≤33 week | 34~35 week | 36 week | 37~39 week |
| bankssts_lh | **6.27 (4.9×10^-5^)** | -3.93 (8.4×10^-5^) | -2.87 (4.0×10^-3^) | -1.24 (0.22) | -1.77 (0.08) |
| fusiform_lh | **7.96 (2.1×10^-6^)** | -5.09 (3.6×10^-7^) | -1.97 (0.05) | -1.32 (0.19) | -2.31 (0.02) |
| inferior parietal_lh | **13.2 (9.6×10^-11^)** | -5.66 (1.6×10^-8^) | -4.58 (4.8×10^-6^) | -2.37 (0.02) | -2.31 (0.02) |
| middle temporal_lh | **14.7 (5.1×10^-12^)** | -6.99 (2.9×10^-12^) | -3.35 (1.0 ×10^-3^) | -1.34 (0.18) | -2.41 (0.02) |
| pars orbitalis_lh | **5.25 (3.0×10^-4^)** | -3.78 (2.0×10^-4^) | -1.81 (0.07) | -2.31 (0.02) | -0.05 (0.96) |
| pars triangularis_lh | **7.24 (8.1×10^-6^)** | -4.27 (2.0×10^-5^) | -2.76 (6.0×10^-3^) | -2.38 (0.02) | 0.09 (0.93) |
| postcentral_lh | **12.8 (2.1×10^-10^)** | -5.54 (3.1×10^-8^) | -2.71 (7.0×10^-3^) | -1.26 (0.21) | -4.51 (6.7×10^-6^) |
| rostral anterior cingulate_lh | **9.17 (2.2×10^-7^)** | -4.79 (1.7×10^-6^) | -3.09 (2.0×10^-3^) | -2.13 (0.03) | -2.78 (5.0×10^-3^) |
| superior parietal_lh | **5.14 (4.0×10^-4^)** | -3.57 (4.0×10^-4^) | -3.03 (2.0×10^-3^) | -1.11 (0.27) | -0.94 (0.35) |
| insula_lh | **6.56 (2.9×10^-5^)** | -3.52 (4.0×10^-4^) | -1.46 (0.14) | -2.14 (0.03) | -3.54 (4.0×10^-4^) |
| bankssts_rh | **8.33 (1.1×10^-6^)** | -4.14 (3.5×10^-5^) | -3.32 (1.0 ×10^-3^) | -2.24 (0.03) | -2.83 (5.0×10^-3^) |
| caudal middle frontal_rh | **6.41 (3.8×10^-5^)** | -4.08 (5.0×10^-5^) | -2.50 (0.01) | 0.43 (0.67) | -2.26 (0.02) |
| fusiform_rh | **7.78 (2.9×10^-6^)** | -4.45 (8.8×10^-6^) | -3.17 (2.0×10^-3^) | -0.89 (0.38) | -2.05 (0.04) |
| inferior parietal_rh | **11.0 (7.0×10^-9^)** | -5.15 (2.6×10^-7^) | -3.69 (2.0×10^-4^) | -1.43 (0.15) | -3.23 (1.0×10^-3^) |
| isthmus cingulate_rh | **5.56 (2.0×10^-4^)** | -4.01 (6.2×10^-5^) | -2.27 (0.02) | -1.64 (0.10) | -1.66 (0.10) |
| middle temporal_rh | **22.5 (1.8×10^-18^)** | -8.35 (7.7×10^-17^) | -4.45 (8.5×10^-6^) | -2.60 (0.01) | -3.06 (2.0×10^-3^) |
| pars orbitalis_rh | **5.90 (1.0×10^-4^)** | -3.74 (2.0×10^-4^) | -2.96 (3.0 ×10^-3^) | -2.24 (0.03) | -0.59 (0.56) |
| pars triangularis_rh | **8.00 (2.0×10^-6^)** | -4.20 (2.6×10^-5^) | -1.86 (0.06) | -3.72 (2.0×10^-4^) | -0.93 (0.36) |
| postcentral_rh | **8.48 (8.0×10^-7^)** | -5.38 (7.7×10^-8^) | -1.68 (0.09) | -1.84 (0.07) | -1.97 (0.05) |
| posterior cingulate_rh | 4.45 (1.0×10^-3^) | -3.29 (1.0×10^-3^) | -2.59 (0.01) | -0.32 (0.75) | -1.40 (0.16) |
| precuneus_rh | 4.62 (1.0×10^-3^) | -3.74 (2.0×10^-4^) | -1.67 (0.10) | -0.93 (0.36) | -1.72 (0.09) |
| superior frontal_rh | **5.05 (5.0×10^-4^)** | -3.77 (2.0×10^-4^) | -0.83 (0.41) | -1.50 (0.13) | -2.50 (0.01) |
| supramarginal_rh | **7.92 (2.3×10^-6^)** | -4.48 (7.6×10^-6^) | -3.28 (0.001) | -2.33 (0.02) | -1.50 (0.13) |
| transverse temporal_rh | 4.69 (9.0×10^-4^) | -3.75 (2.0×10^-4^) | -2.25 (0.03) | -1.04 (0.30) | -1.36 (0.17) |
| insula_rh | **7.58 (4.3×10^-6^)** | -3.84 (1.0×10^-4^) | -1.37 (0.17) | -3.25 (0.001) | -3.10 (2.0×10^-3^) |
| thalamus proper_rh | **11.2 (4.9×10^-9^)** | -6.35 (2.3×10^-10^) | -2.29 (0.02) | -0.72 (0.47) | -1.88 (0.06) |
| pallidum_rh | **4.89 (6.0×10^-4^)** | -3.39 (7.0 ×10^-4^) | -3.09 (2.0 ×10^-3^) | -0.93 (0.35) | -1.01 (0.31) |
| hippocampus_rh | **5.33 (3.0×10^-4^)** | -4.50 (7.0×10^-6^) | -0.48 (0.64) | -0.36 (0.72) | -1.36 (0.17) |
| total left hemisphere | 9.20 (2.1×10^-7^) | -5.01 (5.5×10^-7^) | -2.53 (0.01) | -1.59 (0.11) | -3.13 (2.0×10^-3^) |
| total right hemisphere | 9.29(1.7×10^-7^) | -5.16 (2.5×10^-7^) | -2.50 (0.01) | -1.83 (0.07) | -2.84 (5.0×10^-3^) |
| total brain volume | 9.28(1.75×10^-7^) | -5.10 (3.5×10^-7^) | -2.52 (0.01) | -1.72 (0.09) | -2.99 (3.0×10^-3^) |

Gestational age of more than 40 weeks was taken as the reference group in the simple effect test. All of the displayed *P* values are the original ones. Bold values indicated significance with Bonferroni correction (*P*<0.05). Note: No multiple comparison correction was applied to the simple effect test because it is a post-hoc analysis following the test of main effect. Abbreviations: banksstsrh: banks of superior temporal sulcus; lh, left hemisphere; rh, right hemisphere.

**Table S9.** Group difference for the association between gestational age and brain volume at 9-10 years when excluding adolescents with Caesarian birth

| Volumetric regions | Main Effect  F (*P*) values | Simple Effect  T (*P*) values | | | |
| --- | --- | --- | --- | --- | --- |
|  |  | ≤33 week | 34~35 week | 36 week | 37~39 week |
| bankssts_lh | 4.03 (0.003) | -15.07 (1.7×10^-50^) | -1.37 (0.17) | -2.04 (0.04) | -3.24 (0.001) |
| fusiform_lh | 3.83 (0.004) | -7.2 (6.7×10^-13^) | 0.13 (0.9) | -3.71 (0.0002) | -2.31 (0.02) |
| inferior parietal_lh | **6.49 (3.3×10^-5^)** | -8.8 (1.7×10^-18^) | -6.66 (2.9×10^-11^) | -3.16 (0.0016) | -4.1 (4.2×10^-5^) |
| middle temporal_lh | **9.13 (2.4×10^-7^)** | -11.6 (1.3×10^-30^) | -2.2 (0.03) | -2.23 (0.03) | -1.48 (0.14) |
| pars orbitalis_lh | 2.56 (0.04) | -8.55 (1.5×10^-17^) | 3.4 (0.0007) | -2.49 (0.01) | -2.19 (0.03) |
| pars triangularis_lh | 2.09 (0.08) | -4.18 (3.0×10^-5^) | -2.32 (0.02) | -9.04 (1.9×10^-19^) | 2.49 (0.01) |
| postcentral_lh | **14.13 (1.7×10^-11^)** | -10.85 (3.2 ×10^-27^) | -4.31 (1.6×10^-5^) | -2.01 (0.04) | -10.74 (1.0×10^-26^) |
| rostral anterior cingulate_lh | **5.73 (0.0001)** | -8.46 (3.2×10^-17^) | -4.12 (3.8×10^-5^) | -1.85 (0.06) | -4.19 (2.8×10^-5^) |
| superior parietal_lh | 4.49 (0.001) | -7.51 (6.5×10^-14^) | -1.23 (0.22) | -1.43 (0.15) | -2.35 (0.019) |
| insula_lh | **5.66 (0.0002)** | -6.01 (2.0×10^-9^) | -0.08 (0.94) | -3.33 (0.0009) | -5.75 (9.0×10^-9^) |
| bankssts_rh | **9.4 (1.4×10^-7^)** | -11.66 (3.8×10^-31^) | -8.14 (4.8×10^-16^) | -10.24 (1.9 ×10^-24^) | -7.11 (1.3×10^-12^) |
| caudal middle frontal_rh | 4.18 (0.002) | -8.8 (1.7×10^-18^) | -4.65 (3.4×10^-6^) | 1.78 (0.08) | -2.14 (0.032) |
| fusiform_rh | 4.34 (0.002) | -6.38 (1.9 ×10^-10^) | -0.04 (0.96) | -2.54 (0.01) | -3.49 (0.0005) |
| inferior parietal_rh | **8 (2.0×10^-6^)** | -8.42 (4.4×10^-17^) | -4.73 (2.3×10^-6^) | -3.4 (0.0007) | -5.53 (3.3×10^-8^) |
| isthmus cingulate_rh | **7.16 (9.5×10^-6^)** | -9.79 (1.8×10^-22^) | -8.01 (1.3×10^-15^) | -6.59 (4.9×10^-11^) | -4.36 (1.3×10^-5^) |
| middle temporal_rh | **11.46 (2.8×10^-9^)** | -10.94 (1.2×10^-27^) | -2.35 (0.02) | -2.88 (0.004) | -2.77 (0.006) |
| pars orbitalis_rh | 2.96 (0.02) | -5.72 (1.1×10^-8^) | -2.62 (0.009) | -2.08 (0.04) | -1.26 (0.21) |
| pars triangularis_rh | 4.09 (0.003) | -8.83 (1.4×10^-18^) | 0.58 (0.56) | -11.1 (2.3×10^-28^) | -5.38 (7.7×10^-8^) |
| postcentral_rh | **9.21 (2.0×10^-7^)** | -14.25 (2.0×10^-45^) | -2.39 (0.02) | -4.08 (4.7×10^-5^) | -7.14 (1.0×10^-12^) |
| posterior cingulate_rh | **5.5 (0.0002)** | -11.66 (3.8×10^-31^) | -5.38 (7.6×10^-8^) | -1.08 (0.28) | -0.18 (0.85) |
| precuneus_rh | **5.26 (0.0003)** | -7.76 (9.9×10^-15^) | 2.61 (0.009) | -0.95 (0.34) | -2.34 (0.02) |
| superior frontal_rh | 4.36 (0.002) | -6.53 (7.1×10^-11^) | 2.88 (0.004) | -1.57 (0.12) | -4.63 (3.8×10^-6^) |
| supramarginal_rh | **5.00 (0.0005)** | -7.61 (3.2×10^-14^) | -0.98 (0.33) | -3.02 (0.003) | -4.23 (2.3×10^-5^) |
| transverse temporal_rh | **5.06 (0.0005)** | -15.83 (1.8×10^-55^) | 1.74 (0.08) | -3.79 (0.0002) | -4.22 (2.5×10^-5^) |
| insula_rh | **5.83 (0.0001)** | -6.05 (1.5×10^-9^) | 0.27 (0.79) | -2.75 (0.006) | -4.34 (1.4×10^-5^) |
| thalamus proper_rh | **5.88 (1.0×10^-4^)** | -7.6 (3.6×10^-14^) | -2.08 (0.04) | 1.15 (0.25) | -2.42 (0.02) |
| pallidum_rh | 0.67 (0.61) | -0.89 (0.37) | -2.82 (0.005) | -1.17 (0.24) | 0.3 (0.77) |
| hippocampus_rh | 4.15 (0.002) | -7.56 (4.6×10^-14^) | 1.15 (0.25) | 1.38 (0.17) | 0.41 (0.68) |
| total left hemisphere | 9.41 (1.4×10^-7^) | -7.26 (4.3E-13) | 0.11 (0.91) | -1.57 (0.12) | -3.7 (0.0002) |
| total right hemisphere | 9.52 (1.1×10^-7^) | -7.44 (1.1×10^-13^) | -0.08 (0.94) | -2.07 (0.04) | -3.38 (0.0007) |
| total brain volume | 9.51 (1.2×10^-7^) | -7.35 (2.1×10^-13^) | 0.02 (0.98) | -1.82 (0.07) | -3.54 (0.0004) |

Gestational age of more than 40 weeks was taken as the reference group in the simple effect test. All of the displayed *P* values were the

original ones. Bold values indicated significance with Bonferroni correction (*P*<0.05). Note: No multiple comparison correction was

applied to the simple effect test because it is a post-hoc analysis following the test of main effect. Abbreviations: banksstsrh: banks of

superior temporal sulcus; lh, left hemisphere; rh, right hemisphere.

**Table S10.** Group-by-income interaction for brain volumes in adolescents at 9-10 years

| Volumetric regions | ≤33 week | | | 34~35 week | | | 36 week | | |
| --- | --- | --- | --- | --- | --- | --- | --- | --- | --- |
|  | Low Income | Middle Income | High Income | Low Income | Middle Income | High Income | Low Income | Middle Income | High Income |
| bankssts_lh | 3.10 (0.57) | 3.27 (0.67) | 3.38 (0.63) | 3.25 (0.67) | 3.29 (0.58) | 3.35 (0.60) | 3.26 (0.64) | 3.25 (0.50) | 3.38 (0.61) |
| fusiform_lh | 11.5 (1.69) | 11.8 (1.49) | 12.3 (1.94) | 11.5 (1.88) | 12.1 (1.45) | 12.5 (1.63) | 11.7 (1.50) | 12.2 (1.64) | 12.4 (1.71) |
| inferior parietal_lh | 15.67 (2.36) | 16.0 (2.43) | 16.6 (2.45) | 15.6 (2.44) | 16.4 (2.44) | 16.6 (2.32) | 16.0 (2.62) | 16.3 (2.22) | 16.7 (2.19) |
| middle temporal_lh | 12.5 (1.81) | 13.2 (1.68) | 13.5 (1.92) | 12.9 (2.25) | 13.4 (1.78) | 13.9 (1.76) | 12.9 (1.94) | 13.5 (1.83) | 13.9 (1.83) |
| pars orbitalis_lh | 2.67 (0.36) | 2.76 (0.38) | 2.82 (0.38) | 2.68 (0.39) | 2.80 (0.36) | 2.88 (0.41) | 2.66 (0.39) | 2.76 (0.34) | 2.85 (0.36) |
| pars triangularis_lh | 4.56 (0.84) | 4.66 (0.74) | 4.66 (0.72) | 4.44 (0.663) | 4.79 (0.70) | 4.79 (0.82) | 4.61 (0.80) | 4.69 (0.76) | 4.77 (0.72) |
| postcentral_lh | 11.4 (1.67) | 12.0 (1.78) | 12.2 (1.89) | 11.6 (1.93) | 12.0 (1.67) | 12.6 (1.72) | 11.7 (1.81) | 12.0 (1.72) | 12.6 (1.92) |
| rostral anterior cingulate_lh | 3.11 (0.59) | 3.18 (0.59) | 3.33 (0.61) | 3.11 (0.66) | 3.32 (0.60) | 3.37 (0.58) | 3.11 (0.59) | 3.30 (0.57) | 3.38 (0.63) |
| superior parietal_lh | 15.6 (2.01) | 16.7 (1.83) | 17.4 (2.18) | 16.2 (2.61) | 17.1 (1.86) | 17.1 (2.34) | 16.2 (2.14) | 17.0 (2.17) | 17.3 (2.10) |
| insula_lh | 7.25 (0.94) | 7.46 (0.94) | 7.61 (0.94) | 7.38 (1.02) | 7.56 (0.88) | 7.70 (0.89) | 7.32 (0.78) | 7.44 (0.84) | 7.69 (0.83) |
| bankssts_rh | 2.96 (0.52) | 3.08 (0.54) | 3.08 (0.53) | 3.05 (0.59) | 3.05 (0.46) | 3.15 (0.54) | 2.99 (0.52) | 3.08 (0.49) | 3.17 (0.46) |
| caudal middle frontal_rh | 7.34 (1.51) | 7.47 (1.50) | 8.23 (1.63) | 7.57 (1.78) | 7.91 (1.34) | 8.10 (1.60) | 7.79 (1.52) | 7.93 (1.47) | 8.32 (1.51) |
| fusiform_rh | 11.2 (1.66) | 11.4 (1.52) | 12.2 (1.96) | 11.0 (1.72) | 11.8 (1.53) | 12.1 (1.57) | 11.3 (1.56) | 11.9 (1.70) | 12.1 (1.65) |
| inferior parietal_rh | 19.0 (2.74) | 19.5 (2.78) | 20.2 (3.11) | 19.2 (3.02) | 19.8 (2.43) | 20.4 (2.62) | 19.4 (3.02) | 20.0 (2.67) | 20.4 (2.66) |
| isthmus cingulate_rh | 3.02 (0.63) | 3.02 (0.56) | 3.03 (0.57) | 2.95 (0.54) | 3.10 (0.57) | 3.12 (0.59) | 3.01 (0.64) | 2.98 (0.57) | 3.15 (0.56) |
| middle temporal_rh | 13.6 (1.96) | 14.6 (1.77) | 15.0 (1.99) | 14.1 (2.20) | 14.6 (1.71) | 15.3 (1.86) | 14.2 (1.94) | 14.7 (1.87) | 15.3 (1.90) |
| pars orbitalis_rh | 3.24 (0.49) | 3.33 (0.46) | 3.40 (0.44) | 3.18 (0.43) | 3.40 (0.45) | 3.44 (0.46) | 3.24 (0.46) | 3.38 (0.40) | 3.40 (0.42) |
| pars triangularis_rh | 5.37 (0.96) | 5.50 (1.00) | 5.42 (0.97) | 5.28 (0.77) | 5.54 (0.93) | 5.68 (0.92) | 5.31 (1.00) | 5.47 (0.88) | 5.58 (0.92) |
| postcentral_rh | 10.6 (1.58) | 11.0 (1.49) | 11.4 (1.67) | 10.8 (1.81) | 11.5 (1.61) | 11.6 (1.68) | 10.9 (1.74) | 11.2 (1.71) | 11.6 (1.68) |
| posterior cingulate_rh | 3.99 (0.76) | 4.06 (0.62) | 4.29 (0.83) | 4.00 (0.70) | 4.19 (0.70) | 4.27 (0.72) | 4.09 (0.70) | 4.06 (0.71) | 4.38 (0.74) |
| precuneus_rh | 12.3 (1.81) | 13.0 (1.69) | 13.3 (1.72) | 12.7 (2.05) | 13.1 (1.54) | 13.6 (1.71) | 12.7 (1.66) | 13.0 (1.67) | 13.4 (1.65) |
| superior frontal_rh | 26.6 (3.28) | 27.7 (2.89) | 28.4 (3.38) | 27.2 (3.49) | 28.3 (3.13) | 28.9 (3.18) | 27.4 (3.15) | 27.7 (2.93) | 28.8 (3.22) |
| supramarginal_rh | 12.5 (1.87) | 13.1 (1.71) | 13.2 (1.99) | 12.4 (1.86) | 13.2 (1.75) | 13.5 (1.83) | 12.7 (1.70) | 13.0 (1.84) | 13.6 (1.89) |
| transverse temporal_rh | 1.08 (0.23) | 1.08 (0.22) | 1.13 (0.21) | 1.07 (0.22) | 1.14 (0.21) | 1.17 (0.22) | 1.08 (0.21) | 1.14 (0.19) | 1.18 (0.20) |
| insula_rh | 7.28 (0.91) | 7.48 (0.96) | 7.60 (0.90) | 7.42 (1.09) | 7.59 (0.88) | 7.71 (0.91) | 7.36 (0.90) | 7.44 (0.82) | 7.64 (0.84) |
| thalamus proper_rh | 6.91 (0.76) | 6.96 (0.68) | 7.31 (0.69) | 6.96 (0.81) | 7.16 (0.65) | 7.39 (0.75) | 7.07 (0.69) | 7.30 (0.68) | 7.39 (0.74) |
| pallidum_rh | 1.61 (0.21) | 1.59 (0.20) | 1.66 (0.204) | 1.58 (0.18) | 1.63 (0.18) | 1.65 (0.20) | 1.61 (0.20) | 1.64 (0.20) | 1.66 (0.20) |
| hippocampus_rh | 3.88 (0.51) | 4.01 (0.39) | 4.17 (0.43) | 3.95 (0.51) | 4.11 (0.45) | 4.21 (0.45) | 3.98 (0.40) | 4.11 (0.43) | 4.18 (0.43) |
| total left hemisphere | 279.9 (29.7) | 292.0 (26.4) | 301.6 (29.8) | 283.7 (34.0) | 296.7 (23.7) | 303.8 (26.4) | 285.8 (29.0) | 294.1 (27.1) | 303.1 (26.9) |
| total right hemisphere | 280.8 (29.6) | 292.9 (26.4) | 301.6 (29.5) | 284.3 (33.8) | 297.5 (24.1) | 304.6 (26.1) | 286.5 (28.9) | 294.1 (26.7) | 303.5 (26.7) |
| total brain volume | 560.6 (59.2) | 584.9 (52.8) | 603.2 (59.3) | 568.0 (67.8) | 594.1 (47.7) | 608.5 (52.4) | 572.3 (57.7) | 588.3 (53.7) | 606.65 (53.5) |

**Table S10.** Group-by-income interaction for brain volumes in adolescents at 9-10 years (continued)

| Volumetric regions | 37~39 week | | | ≥40 week | | | Interaction | |
| --- | --- | --- | --- | --- | --- | --- | --- | --- |
|  | Low Income | Middle Income | High Income | Low Income | Middle Income | High Income | F values | *P* values |
| bankssts_lh | 3.36 (0.62) | 3.27 (0.55) | 3.37 (0.58) | 3.27 (0.58) | 3.35 (0.58) | 3.43 (0.60) | 1.86 | 0.06 |
| fusiform_lh | 12.1 (1.65) | 12.3 (1.73) | 12.4 (1.63) | 11.87 (1.68) | 12.4 (1.67) | 12.7 (1.68) | 0.47 | 0.88 |
| inferior parietal_lh | 16.5 (2.52) | 16.2 (2.40) | 16.8 (2.26) | 16.3 (2.23) | 16.9 (2.33) | 17.0 (2.30) | 0.87 | 0.54 |
| middle temporal_lh | 13.5 (1.99) | 13.3 (1.83) | 13.9 (1.66) | 13.1 (1.79) | 13.8 (1.81) | 14.1 (1.82) | 0.80 | 0.61 |
| pars orbitalis_lh | 2.79 (0.37) | 2.86 (0.38) | 2.87 (0.34) | 2.72 (0.39) | 2.83 (0.38) | 2.89 (0.37) | 1.79 | 0.08 |
| pars triangularis_lh | 4.69 (0.71) | 4.76 (0.80) | 4.91 (0.79) | 4.68 (0.76) | 4.81 (0.76) | 4.84 (0.78) | 1.85 | 0.06 |
| postcentral_lh | 11.9 (1.83) | 12.0 (1.69) | 12.3 (1.55) | 11.8 (1.77) | 12.3 (1.76) | 12.7 (1.79) | 1.85 | 0.06 |
| rostral anterior cingulate_lh | 3.24 (0.50) | 3.28 (0.55) | 3.35 (0.53) | 3.20 (0.59) | 3.34 (0.60) | 3.42 (0.60) | 1.79 | 0.08 |
| superior parietal_lh | 16.8 (2.01) | 17.0 (2.15) | 17.4 (2.04) | 16.4 (2.26) | 17.1 (2.24) | 17.4 (2.22) | 0.67 | 0.72 |
| insula_lh | 7.49 (0.85) | 7.49 (0.99) | 7.59 (0.88) | 7.41 (0.86) | 7.67 (0.86) | 7.73 (0.85) | 1.60 | 0.12 |
| bankssts_rh | 3.12 (0.52) | 3.03 (0.51) | 3.17 (0.52) | 3.07 (0.50) | 3.18 (0.51) | 3.24 (0.51) | 1.25 | 0.27 |
| caudal middle frontal_rh | 7.72 (1.39) | 7.85 (1.64) | 8.17 (1.59) | 7.65 (1.52) | 8.15 (1.58) | 8.24 (1.54) | 1.38 | 0.20 |
| fusiform_rh | 11.7 (1.65) | 11.8 (1.58) | 12.1 (1.53) | 11.5 (1.58) | 12.0 (1.61) | 12.2 (1.64) | 2.06 | 0.04 |
| inferior parietal_rh | 19.9 (2.62) | 19.7 (2.67) | 20.4 (2.57) | 19.7 (2.61) | 20.4 (2.66) | 20.7 (2.61) | 1.53 | 0.14 |
| isthmus cingulate_rh | 3.13 (0.60) | 3.05 (0.58) | 3.17 (0.56) | 3.07 (0.58) | 3.18 (0.59) | 3.20 (0.57) | 2.00 | 0.04 |
| middle temporal_rh | 14.8 (1.88) | 14.7 (1.88) | 15.3 (1.68) | 14.4 (1.89) | 15.2 (1.92) | 15.5 (1.86) | 0.96 | 0.47 |
| pars orbitalis_rh | 3.36 (0.48) | 3.40 (0.48) | 3.46 (0.47) | 3.30 (0.46) | 3.43 (0.45) | 3.48 (0.45) | 1.44 | 0.18 |
| pars triangularis_rh | 5.40 (0.87) | 5.59 (0.90) | 5.74 (0.88) | 5.53 (0.92) | 5.65 (0.91) | 5.66 (0.93) | 0.49 | 0.87 |
| postcentral_rh | 11.4 (1.77) | 11.2 (1.60) | 11.6 (1.48) | 11.0 (1.68) | 11.5 (1.70) | 11.7 (1.67) | 0.35 | 0.95 |
| posterior cingulate_rh | 4.24 (0.85) | 4.19 (0.75) | 4.28 (0.79) | 4.14 (0.74) | 4.26 (0.71) | 4.32 (0.71) | 0.90 | 0.52 |
| precuneus_rh | 12.8 (1.65) | 13.2 (1.88) | 13.5 (1.56) | 12.7 (1.74) | 13.3 (1.70) | 13.5 (1.67) | 1.17 | 0.31 |
| superior frontal_rh | 27.7 (3.25) | 27.6 (3.19) | 28.9 (2.92) | 27.3 (3.21) | 28.4 (3.23) | 29.0 (3.15) | 0.95 | 0.47 |
| supramarginal_rh | 13.0 (1.97) | 13.2 (1.95) | 13.6 (1.74) | 12.9 (1.98) | 13.5 (2.03) | 13.6 (2.00) | 0.45 | 0.90 |
| transverse temporal_rh | 1.13 (0.21) | 1.15 (0.21) | 1.17 (0.22) | 1.11 (0.21) | 1.17 (0.22) | 1.18 (0.21) | 1.35 | 0.21 |
| insula_rh | 7.53 (0.90) | 7.42 (0.97) | 7.67 (0.90) | 7.43 (0.88) | 7.68 (0.89) | 7.74 (0.89) | 1.20 | 0.30 |
| thalamus proper_rh | 7.21 (0.78) | 7.18 (0.75) | 7.377 (0.73) | 7.13 (0.68) | 7.32 (0.69) | 7.35 (0.68) | 1.16 | 0.32 |
| pallidum_rh | 1.65 (0.22) | 1.66 (0.22) | 1.67 (0.193) | 1.63 (0.20) | 1.66 (0.20) | 1.68 (0.20) | 1.43 | 0.18 |
| hippocampus_rh | 4.02 (0.45) | 4.05 (0.42) | 4.18 (0.42) | 3.98 (0.42) | 4.12 (0.42) | 4.17 (0.42) | 2.03 | 0.04 |
| total left hemisphere | 292.5 (28.0) | 292.7 (28.2) | 302.9 (24.5) | 287.3 (28.0) | 300.0 (27.8) | 304.9 (27.2) | 1.08 | 0.38 |
| total right hemisphere | 293.7 (27.9) | 294.1 (28.8) | 303.9 (24.2) | 288.2 (28.1) | 300.7 (27.9) | 305.6 (27.1) | 1.78 | 0.08 |
| total brain volume | 586.2 (55.8) | 586.8 (56.9) | 606.8 (48.6) | 575.5 (55.9) | 600.6 (55.5) | 610.5 (54.2) | 1.71 | 0.09 |

The values represented mean (std) cm^3^. Abbreviations: banksstsrh: banks of superior temporal sulcus; lh, left hemisphere; rh, right hemisphere.


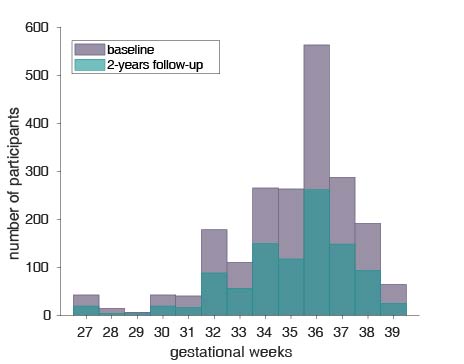


**Figure S1.** **The distribution of gestational weeks at both baseline and 2-years follow-up in neuroimaging analyses.**


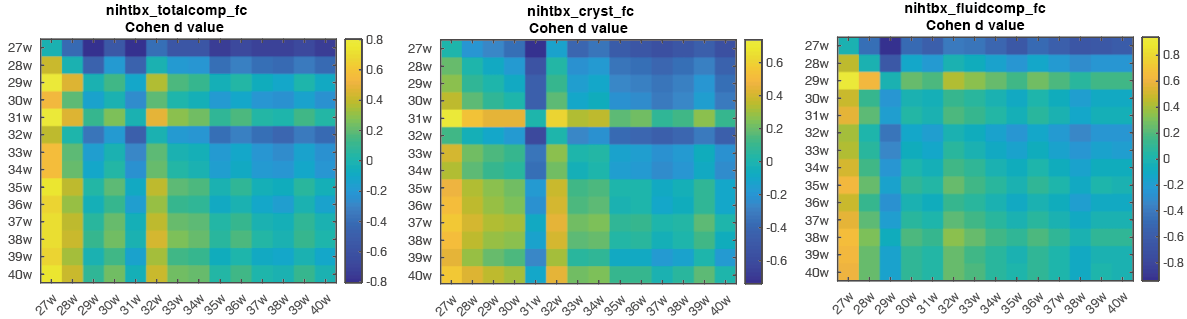


**Figure S2. Differences of cognitive performance between any two gestational weeks.** nihtbx_totalcomp_fc: Cognition Total Composite Score Fully-Corrected T-score; nihtbx_cryst_fc: Crystallized Composite Fully-Corrected T-score; nihtbx_fluidcomp_fc: Cognition Fluid Composite Fully-Corrected T-score.

**
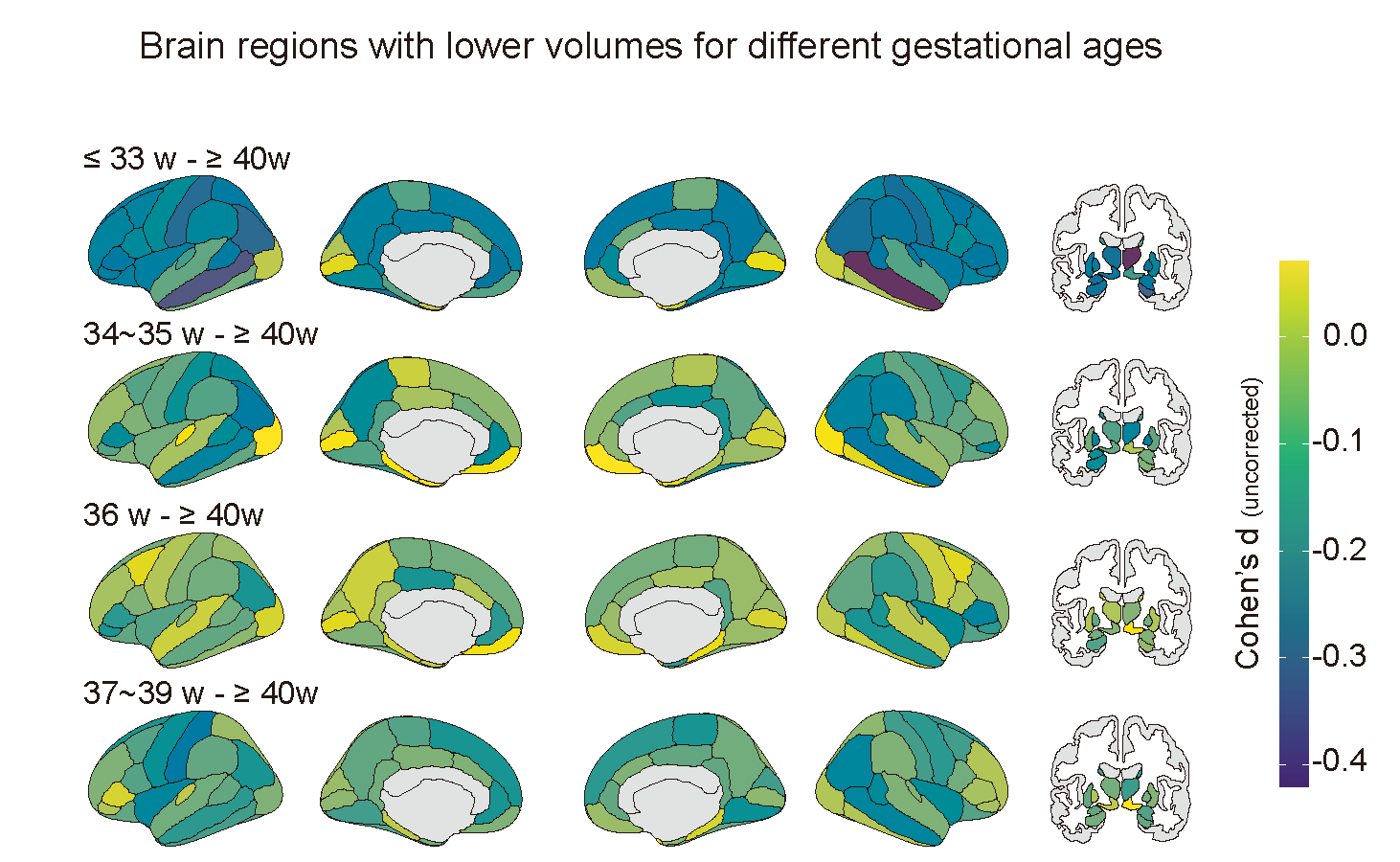
**

**Figure S3.** **Brain regions with lower volumes for different gestational age in 11,203 adolescents at baseline. Pairwise comparisons of the volumes of brain regions for adolescents in each group of gestational age and those at 40 weeks without a statistical threshold. The color represents the Cohen’s d value. Similar brain map with Bonferroni correction (*P*<0.05) was shown in Figure 3B in the main text. The surface visualization was generated using the R package ggseg (https://github.com/ggseg/ggseg).**

**
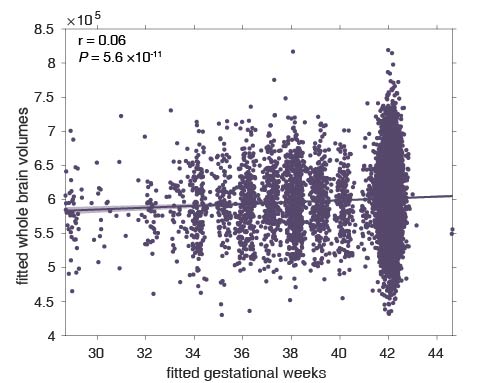
**

**Figure S4.** **Positive correlation between gestational age and whole brain volumes at baseline.**


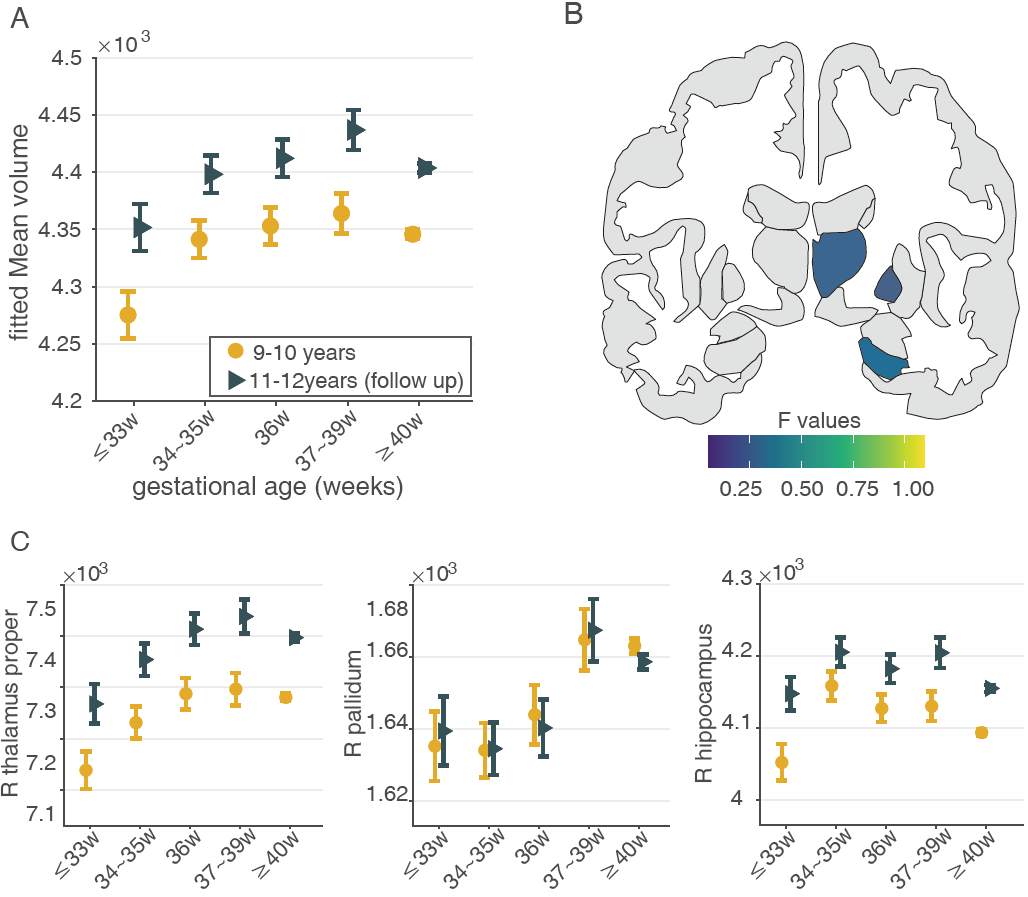


**Figure S5.** **Subcortical/non-neocortical volume growth from 9-10 years to 11-12 years.** **A.** Volumetric growth trajectories for the non-neocortical/subcortical regions that have been shown significant group difference at baseline. **B.** No significant interaction of group-by-time for each region, including the right thalamus, right pallidum and right hippocampus. The color represents F value. **C.** Volumetric growth trajectories for each subcortical/non-neocortical region. Abbreviations: R, Right.


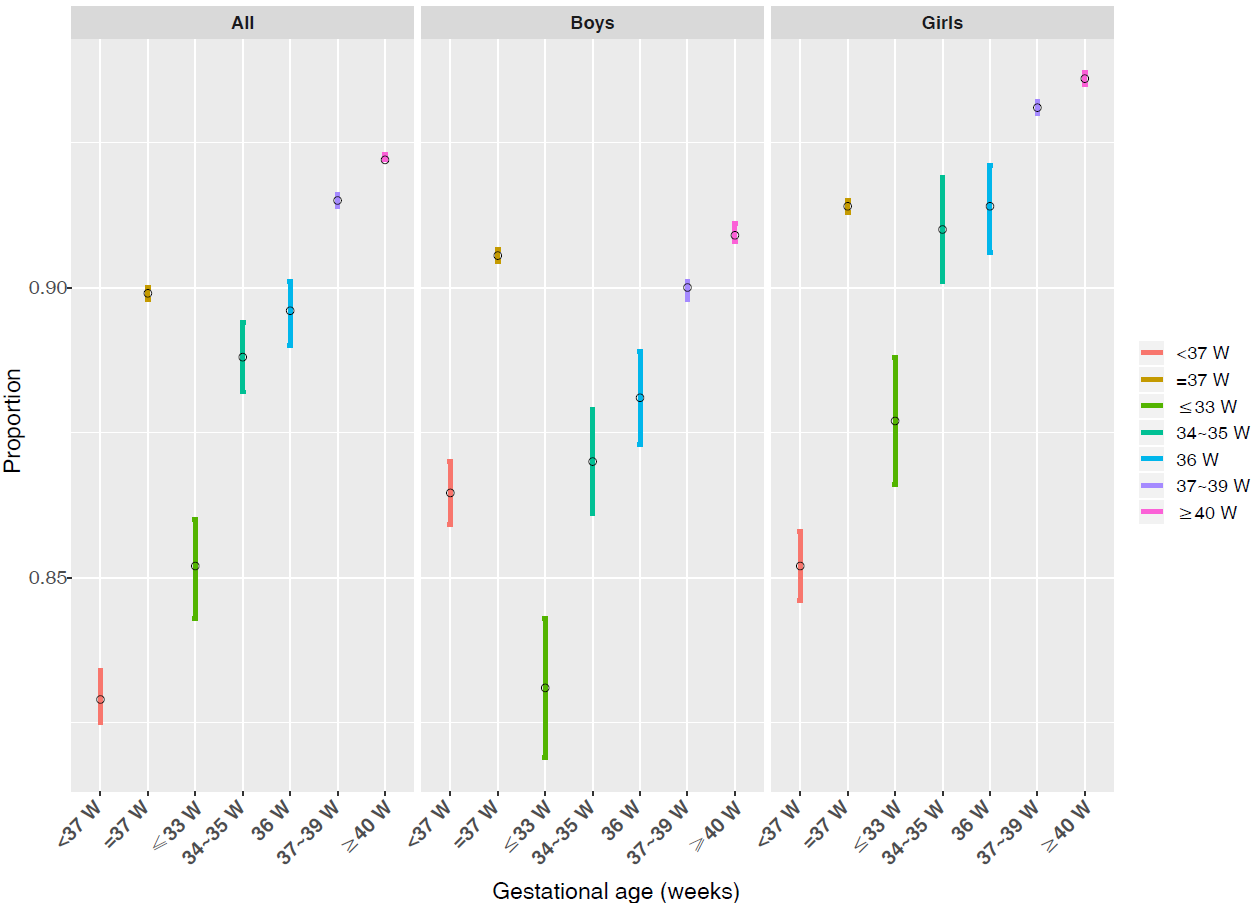


**Figure S6**. **Proportion of adolescents taking the final examination according to gestational weeks from the Danish cohort study.**
